# Probability maps of anthropogenic impacts affecting ecological status in European rivers

**DOI:** 10.1016/j.ecolind.2021.107684

**Published:** 2021-07

**Authors:** Olga Vigiak, Angel Udias, Alberto Pistocchi, Michela Zanni, Alberto Aloe, Bruna Grizzetti

**Affiliations:** a European Commission, Joint Research Centre (JRC), via E Fermi 2749, 21020 Ispra, VA, Italy; b ARHS Developments Italia S.r.l., Via F.lli Gabba 1/A, 20121 Milano, Italy; c ARHS Developments, 13 Boulevard du Jazz, L-4370 Belvaux, Luxembourg

**Keywords:** Water Framework Directive, Aquatic habitat, Ecological status, River impact, Anthropogenic pressures

## Abstract

Understanding how anthropogenic pressures affect river ecological status is pivotal to designing effective management strategies. Knowledge on river aquatic habitats status in Europe has increased tremendously since the introduction of the European Union Water Framework Directive, yet heterogeneities in mandatory monitoring and reporting still limit identification of patterns at continental scale. Concurrently, several model and data-based indicators of anthropogenic pressures to freshwater that cover the continent consistently have been developed. The objective of this work was to create European maps of the probability of occurrence of river conditions, namely failure to achieve good ecological status, or to be affected by specific pervasive impacts. To this end, we applied logistic regression methods to model the river conditions as functions of continental-scale water pressure indicators. The prediction capacity of the models varied with river condition: the probability to fail achieving good ecological status, and occurrence of nutrient and organic pollution were rather well predicted; conversely, chemical (other than nutrient and organic) pollution and alteration of habitats due to hydrological or morphological changes were poorly predicted. The most important indicators explaining river conditions were the shares of agricultural and artificial land, mean annual net abstractions, share of pollution loads from point sources, and the share of upstream river length uninterrupted by barriers. The probability of failing to achieve good ecological status was estimated to be high (>60%) for 36% of the considered river network of about 1.6 M km. Occurrence of impact of nutrient pollution was estimated high (>60%) in 26% of river length and that of organic pollution 20%. The maps are built upon information reported at country level pursuant EU legal obligations, as well as indicators generated from European scale models and data: both sources are affected by epistemic uncertainty. In particular, reported information depend on data collection scoping and schemes, as well as national knowledge and interpretation of river system pressures. In turn, water pressure indicators are affected by heterogeneous biases due to incomplete or incorrect inputs and uncertainty of models adopted. Lack of effective reach- and site-scale indicators may hamper detection of locally relevant impacts, for example in explaining alteration of habitats due to morphological changes. The probability maps provide a continental snapshot of current river conditions, and offer an alternative source of information on river aquatic habitats, which may help filling in knowledge gaps. Foremost, the analysis demonstrates the need for developing more effective continental-scale indicators for hydromorphological alterations and chemical pollution.

## Highlights

• Probability of river impacts occurrences were estimated from pressure indicators.
• Good ecological status failure, nutrient and organic pollution were well predicted.
• Probability to fail achieving good ecological status was >60% in 36% of river length.
• Hydro-morphological alteration mapping requires specific water pressure indicators.
• The maps of impact occurrence probability are useful for river basin management.

## Graphical abstract

**Figure:**
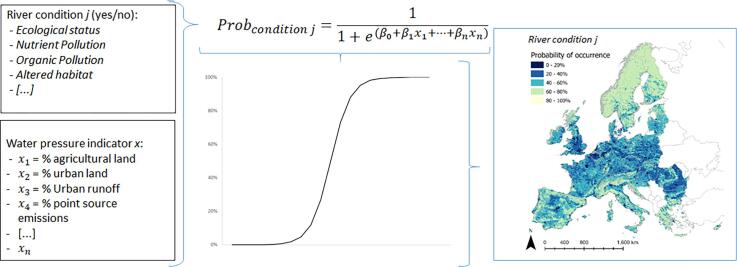

## 1 Introduction

In 2000 the Water Framework Directive (WFD; EC, 2000) provided the environmental legislative framework to safeguard water resources in the European Union, setting the target of achieving (or maintaining) good ecological status in all EU rivers, lakes, transitional, and coastal waters through River Basin Management Plans (RBMPs). Fifteen years after its entry into force, the second round of RBMPs, referring to condition for 2010–2015, indicates that rivers achieving at least good ecological status are about 40% (EEA, 2020a). The main reasons for failing to meet good status comprise the many challenges posed by managing increasing demands of water resources while preserving habitats condition, the multiplicity of pressures acting on these systems, and the time necessary for ecosystems to recover (e.g. Carvalho et al., 2019, Birk et al., 2020, Santos et al., 2021, Lemm et al., 2021).

The WFD allowed remarkable progress in water management in the EU, not least because it enabled setting a consistent framework across European countries to monitor and report water bodies ecological and chemical status as well as to identify main anthropogenic drivers and impacts that has much improved our knowledge of aquatic habitat condition and pressures. In the second RBMP reporting round, the knowledge basis for assessing status and impacts has much improved compared to the first round; the number of water bodies in unknown ecological status has significantly decreased, while comparability of monitoring methods has improved through intercalibration efforts (Poikane et al., 2014, European Environment Agency (EEA). 2018. European waters - Assessment of status and pressures, 2018, Lyche Solheim et al., 2019, Santos et al., 2021). Additionally, the latest reporting round provides information on the pressures and impacts that mostly affect water bodies. Despite these major steps forward, some knowledge gaps remain. For example, the ecological status of rivers is declared ‘Unknown’ in 5% of river water bodies (Supportive information, Fig SI1). Furthermore, heterogeneity in reporting is still evident. For example, methods used to assess ecological status differ for their capacity to respond to key pressures and in adopted assessment criteria (Poikane et al., 2019, Poikane et al., 2020, European Environment Agency (EEA), 2019, Santos et al., 2021). National reporting, especially when done in the form of presence-absence, is affected by monitoring scheme scoping and implementation, regional variability of pressures, and local perceptions of links between impacts and ecological status (Fluet-Chouinard et al., 2020). These issues still prevent attaining homogeneous continental snapshots of freshwater condition; yet, spatial patterns are useful to identify regions of prior intervention and, when put in relation to anthropogenic pressures, may provide insights into pressure-impact and pressure-status relationships that can inform planning of management strategies.

Concurrently, Europe-wide indicators that help assess pressures on aquatic habitats have been developed from continental scale data and models (Pistocchi et al., 2015, Pistocchi et al., 2017, Pistocchi et al., 2018). These indicators may help mapping out freshwater pressures evenly and filling in potential knowledge gaps. Using several statistical approaches, Grizzetti et al. (2017) showed that the water pressure indicators can be related to the ecological status of river network (Grizzetti et al., 2017). Since Grizzetti et al. (2017) study, the water pressure indicator suite has been revised and expanded. Particularly, net abstractions, low flow alteration, nitrogen and phosphorus concentration have been recently updated (Bisselink et al., 2018, Grizzetti et al., Submitted for publication), whereas an indicator of organic pollution, the mean annual concentration of 5-days Biochemical Oxygen Demand (BOD, mg/L) has been added (Vigiak et al., 2019). The improved indicator suite, together with the enhanced quality of the WFD reporting data, provides new venues for mapping river conditions at continental scale, and exploring not only their relationship with ecological status but also with single impact types.

The aim of the analysis was to produce European maps of the probability of occurrence of certain river conditions, namely to fail meeting good ecological status, or to be affected by specific impacts. These conditions were modelled as functions of continental-scale water pressure indicators in order to (i) provide a coherent continental snapshot of river aquatic habitats and (ii) fill in knowledge gaps. Further, we wanted to quantify the marginal effects of water pressure indicators on the river impacts to improve the understanding of multiple pressure-impact and pressure-status relationships in freshwater systems. In the remainder of the paper, we present the data used for the analysis and the methods applied to derive the probability maps, we present and discuss the results, providing conclusive messages on their expected use in support to the implementation of the EU Water Framework Directive.

## 2 Material and methods

### 2.1 River conditions (ecological status and impacts) and anthropogenic pressure indicators

The WFD defines the ecological status (ES) of water bodies depending on the deviation from their undisturbed conditions. The ES is a synthetic judgment on the state of a water body, based on a range of biological, hydrological and water quality elements. The RBMPs must include a classification of the ES for all water bodies subject to the WFD, and pursue the general objective of achieving good ES for all water bodies, unless otherwise justified.

The ES of all river water bodies is reported by the Member States to the European Union and other European countries according to article 13 of the Water Framework Directive (WFD) and compiled in a geo-spatial dataset, the WISE-WFD database (from now on WISE; EEA, 2020a). This contains information about surface and ground water bodies, but in our study we focus on rivers only. WISE provided information for 114,125 river water bodies reported for the 2nd RBMP round with reference year 2016 for condition in 2010–2015. Of all the river water bodies, only 100,702 could be associated with geographic data (georeferenced streamlines; EEA, 2020b). The ES is reported in classes (high, good, moderate, bad, and poor), or Unknown (see supporting information, Figure SI1). Furthermore, the database reports binary information (1 = presence or 0 = absence) of impact types acting on the water body. The reported presence of an impact type means that this impact, alone or in combination with others, puts achieving environmental objectives (good chemical and ecological status) at risk (EEA, 2016). Reported impact types in rivers varied between countries but mainly comprised chemical pollution (reported in 37% of rivers, Table 1), nutrient (26%) and organic (19%) pollution, altered habitats due to hydrological (14%) or morphological (35%) changes. Conversely, acidification (5%), microbiological pollution (1%), salinity or other intrusions (1%), high temperature (1%), other (2%) and unknown (10%) impacts were less frequently reported. The absence of any impact (type “None”; EEA, 2016) was reported in 29% of river water bodies. Finally, very few cases reported litter or unclear (i.e. unexplained in the glossary) impacts. Any impact type can be reported for each water body, therefore the number of impact types per river water body varied from zero to more than five (Fig. SI1).

**Table 1:** Number of river water bodies reported in WISE-WFD database, and percentage of rivers affected by impact types (summarized from EEA, 2020a, second RBMPs reporting round). In the last two columns the number of river water bodies used in the analysis is reported based on the fraction of water body comprised in the paired CCM2 catchments (“wb > 50” = at least 50% of the water body falls in its paired CCM2 catchment; “wb < 50” = <50% of the water body falls in its paired CCM2 catchment).

Country	River water bodies	Chemical Pollution	Altered Morphology	Nutrient Pollution	Organic Pollution	Altered Hydrology	Any other impacts	No impact (None)	Main other impact	Rivers in “wb<50” group	Rivers in “wb>50” group
	n	%								n	n
AT	8065	100	43	18	2	17	1	0	“Unknow”	3871	2147
BE	527	39	6	64	34	0	37	0	“Unknown”	259	201
BG	873	5	6	39	23	3	21	38	“Unknown”	238	535
CY	174	0	29	18	14	0	14	44	“Salinity”	84	30
CZ	1044	50	0	41	20	0	23	14	“Unknown”	469	556
DE	8998	99	93	78	32	12	2	0	“Salinity”	3962	2530
DK	7776	0	38	0	55	0	9	21	“Unknown”	949	101
EE	645	1	24	7	0	1	68	0	“Unknown”	298	233
EL	1345	14	7	17	15	7	20	61	“Unknown”	471	645
ES	4390	17	40	34	27	26	20	35	“Other”	1437	2634
FI	1913	33	15	39	16	7	12	44	“Other”	1083	741
FR	10706	38	41	32	28	26	12	31	“Unknown”	5160	4192
HR	1484	8	19	44	24	18	3	41	“Unknown”	768	476
HU	963	8	88	43	13	8	22	3	“Salinity”	422	372
IE	3192	1	19	28	11	6	24	44	“Unknown”	1174	688
IT	7493	26	23	26	26	18	29	30	“Unknown”	2483	3089
LT	822	1	16	29	6	4	4	49	Unclear	335	397
LU	110	100	99	90	100	7	0	0		75	31
LV	203	9	42	57	0	2	13	20	“Unknown”	47	140
MT	3	0	0	0	0	0	100	0	“Unknown”	0	0
NL	246	54	77	84	50	72	48	0	“Other”	83	51
NO	19532	4	11	17	12	10	28	64	“Unknown”	5276	5422
PL	4586	6	10	12	5	0	46	36	“Unknown”	2088	2093
PT	1899	4	8	27	41	8	10	52	“Unknown”	916	642
RO	2891	2	1	27	18	4	7	65	“Unknown”	1450	1334
SE	15092	100	59	8	0	29	16	0	“Acidification”	9712	1861
SI	137	100	28	73	76	23	0	0		22	107
SK	1510	6	35	13	27	0	55	0	“Unknown”	832	589
UK	7506	4	32	36	15	11	21	29	“Other”	0	0
TOTAL	114125	37	35	26	19	14	18	29		43965	31837

In order to model the spatial distribution of reported conditions (ES and impact types) of European rivers, we use the JRC Water Pressure Indicator dataset (WPI), a collection of indicators developed with European-scale models and data to help assess the distribution of the most frequent anthropogenic pressures in European freshwaters (Pistocchi et al., 2015, Pistocchi et al., 2017, Pistocchi et al., 2018; updated and expanded with Bisselink et al., 2018, Grizzetti et al., Submitted for publication; Vigiak et al., 2019). The dataset comprises indicators for flow regime alteration, nutrient pollution, urban runoff, morphological alterations in riparian land, and presence of barriers along the stream network. All indicators were developed based on European-wide data and models. Except for abstractions and low flow alteration, indicators were assessed at the spatial resolution and extent of the CCM2 River and Catchment Database for Europe (Vogt et al., 2007, Vogt et al., 2008, De Jager and Vogt, 2007), for catchments of mean area of 6.4 km^2^, each with one main associated river reach. Net abstractions and low flow alteration were originally provided as a raster of 5 × 5 km pixel size (Bisselink et al., 2018; Fig. SI4). For this study, these data were resampled at the CCM2 catchment scale. A selection of WPI indicators (Table 2) was used to explain (explanatory variables) the distribution of reported river conditions. The selection was driven by the need to pick indicators that could represent as many anthropogenic pressures as possible while keeping the number of explanatory variables and their correlations to a minimum (Fig. SI5). In addition, the mean annual rainfall was included in the explanatory variable set to take in consideration the main climatic gradient.

**Table 2:** Explanatory variables included in the analysis: mean rainfall and Water Pressure Indicators (WPI).

Acronym	Unit of measure	Definition	Reason for inclusion
Rain	mm	Mean annual rainfall (mm) in 2005–2012 in the drainage area.	Climate; hydrology
ArtiArea	Fraction	Area of urban/artificial land in the catchment divided by the catchment area. The area was based on Corine CLC 2012 dataset	Importance of urban land in the catchment
AgriArea	Fraction	Area of agricultural land in the catchment divided by the catchment area. The area was based on Corine CLC 2012 dataset	Importance of agriculture in the catchment
TN_mgL	mg N/l	Mean annual nitrogen concentration in rivers in 2005–2012. Estimated with GREEN model (Grizzetti et al., 2017, Grizzetti et al., Submitted for publication)	Nutrient pollution
NShareAgri	Fraction	Share of total nitrogen load due to agriculture; estimated with GREEN model (Grizzetti et al., 2017, Grizzetti et al., Submitted for publication).	Contribution to nutrient loads from agriculture
NSharePoint	Fraction	Share of total nitrogen load due to point sources; estimated with GREEN model (Grizzetti et al., 2017, Grizzetti et al., Submitted for publication).	Contribution to nutrient loads from point sources
UrbRunoffShare	Fraction	Fraction of urban runoff in river flow	Contribution to flow and pollution from urban land
BOD_mgL	mg O2/l	Mean annual BOD concentration (mg/L; Vigiak et al., 2019)	Organic pollution
Abstractions	mm	Estimated net water abstraction in the reach drainage area (Bisselink et al., 2018; period reference 2000–2018)	Water abstractions, over-exploitation
Q10	Fraction	Frequency with which each annual flow is below the natural 10th flow percentile. It is a measure of low flow alteration due to water abstractions, the higher the value the higher the alteration (Bisselink et al., 2018; period reference 2000–2018)	Hydrological alteration
NaturalFlood_RipFr	Fraction	Fraction of floodplain areas occupied by natural land cover (Pistocchi et al., 2018)	Potential purification in riparian/floodplains areas; also, the potential presence of morphological alterations in floodplains and riparian areas
DamFreeLength	Fraction	Fraction of stream network length accessible between consecutive stream barriers. For each catchment reach, the indicator is computed as the total extent of the aquatic habitat that a swimming organism can potentially access in the presence of stream barriers, divided by the total habitat extent without barriers. The closer to 0, the more fragmented the habitat; the closer to 1, the more natural (Pistocchi et al., 2018).	Impact of barriers on longitudinal connectivity

### 2.2 Methodology and binary logistic regression models

The methodology comprised the following steps: 1) definition of general regression modelling approach; 2) spatial intersection of geodatasets; 3) preparation of balanced training and validation samples; 4) model training and validation; and 5) generation of European maps.

The relationships between reported river conditions and the explanatory variables were analyzed through logistic regression models. Binary Logistic Regression is a statistical model used to explain the relationship between one binary dependent variable and multiple explanatory variables, which can be ordinal, nominal, interval, and ratio-scale. The binary dependent variable is transformed into a logit variable representing the natural logarithm of the odds of the dependent occurring or not (Atkinson and Massari, 1998, Podgorski et al., 2017). The generic form is:

(1) $$p_{c}=\frac{1}{1+e^{\left(\beta_{0}+\beta_{1}x_{1}+\cdots +\beta_{n}x_{n}\right)}}$$

where *p_c_* = probability of the condition *c* to occur (0–1), β_0_ is the intercept, β_1_ to β_n_ are coefficients for the *n* explanatory variables *x*. The coefficients are estimated with maximum likelihood inference given the training sample. Logistic regressions have been applied successfully to map environmental issues (Nolan et al., 2002, Podgorski et al., 2017). While other machine learning methods, like Regression Trees or Random Forest may often result in higher predictive performance (e.g. Grizzetti et al., 2017, Glendell et al., 2019), logistic regressions maintain an explicit effect of explanatory variables on the probability outcome, i.e. the marginal effect, thus their interpretation is more straightforward than for machine learning approaches. Given the interest in quantifying the marginal effects of water pressure indicators on river impacts, we opted for logistic regressions over other approaches.

The main river conditions reported in WISE-WFD were used as binary response variables *c*. The analysis included: (i) probability of failing to achieve good ecological status; (ii) probability of occurrence of nutrient, organic, and chemical pollution, of altered habitats due to morphological or hydrological changes; and (iii) probability of no impact occurrence. To model failing to achieve good ecological status, ES classes high or good were merged (class 0) whereas classes moderate, poor or bad where classified as 1. Less frequently reported impact types could not be included because of imbalanced cases of presence vs absence.

The set of explanatory variables comprised 12 variables (Table 2). To quantify the marginal effect of water pressure indicators, the explanatory variables were not standardized. This choice did not affect the predictive capacity of the models. Model fitting of logistic regression is however sensitive to collinearities among explanatory variables (Hosmer and Lemeshow, 1989, Podgorski et al., 2017). Generally, a variance inflation factors (VIF) > 5 and a tolerance < 0.1 indicate multi-collinearity (Johnston et al., 2018). For each condition, the number of explanatory variables was reduced in a stepwise process considering simultaneously variable collinearities and importance (using Akaike Information Criterion - AIC; Akaike, 1974).

### 2.3 Spatial intersection of datasets.

The two geo-spatial datasets differ for geographic features and content; their association was based on the spatial intersection of the geographical features of WISE water bodies (EEA, 2020b) with CCM2 catchments. Oversea territories and Iceland were excluded from the analysis as CCM2 does not cover them, whereas the UK was excluded because no WISE geographic information is available for it.

The spatial intersection yielded several combinations of WISE-CCM2 pairs; for example, when a WISE water body was split into several CCM2 catchments, or when more than one WISE water body fell in the same CCM2 catchment. To avoid redundancy in the statistical analysis, from all combinations only one WISE water body-CCM2 catchment pair was retained for further analysis, choosing the CCM2 catchment that contained the longest (or the largest when WISE river water bodies were reported as polygons) fraction of WISE water body. Inevitably, some WISE water bodies were excluded, for example when several small water bodies fell in the same CCM2 catchment. The procedure led to an abridged dataset of 82,178 pairs where each CCM2 and each WISE water body appeared in the final dataset only once. Finally, among these, there were cases of incomplete data: either the ES was unknown (Figure SI1a) or some of the WPI were missing, e.g. in some coastal catchments where the CCM2 catchments had no associated reach, whose discharge and concentrations were undefined. Incomplete data cases were excluded, leaving 75,801 WISE-CCM2 pairs retained for the analysis.

This dataset of WISE-CCM2 pairs was divided in two groups, based on the fraction of the original WISE water body that fell in its associated catchment. The first group consisted of all WISE-CCM2 pairs where this fraction was equal or >50% (“wb > 50”). This group comprised 43,964 data entries (58% of dataset). To maximize the probability that explanatory variables, which are defined per CCM2 catchment, were representative of the WISE water body associated to it, samples for model training were drawn only from this group. The remaining 31,837 WISE-CCM2 pairs, i.e. where the WISE water body segment associated to the CCM2 reach was <50% of the water body (“wb < 50” group), were used for model validation.

Some heterogeneity in the spatial distribution of the “wb > 50” and the “wb < 50” groups was generated because of how countries delineated the water bodies in relation to the CCM2 layer (Table 1; Figure SI2). For example, some countries defined river water bodies as very small reaches (e.g. DK), while others defined very long rivers, or groups of reaches (e.g. BG). Generally, shorter river water bodies tended to be included in the “wb > 50%” group whereas long water bodies were more represented in the “wb < 50%” group (Table 1; Figure SI2). Despite the spatial heterogeneity, a comparison between other characteristics of rivers included in either group showed that the “wb > 50” group was representative of the river population in terms of ecological status, water body type, Strahler order, mean rainfall, and drainage area (Fig. SI3).

### 2.4 Preparation of training and validation samples

In binary classification predictive modeling, extraction of a representative sample is crucial for obtaining valid and robust results, therefore particular care was taken in drawing representative and balanced samples for logistic regressions training. First of all, samples for model training were drawn exclusively from the “wb > 50” group to ensure that the CCM2 explaining variables could represent the water body condition. Second, samples were to be balanced for positive or negative cases (e.g. Salas-Eljatib et al., 2018). Third, it was important that all countries were included in the sample and that entries from any single country would not dominate model training.

A random sampling selection (RSS) was performed to select the training sets (one for each reported river condition) that fulfilled two conditions, namely that (i) it would be composed by an equal number of presence and absence of the condition, and (ii) the maximum number of data from a single country did not exceed the total number of data for the most abundant class (presence or absence) divided by the number of countries. Since the number of water bodies included in the “wb > 50” group varied among countries and the impacts changed with each country (Table 1), the size and composition of WISE-CCM2 pairs in the training sets changed for each river condition model. The RSS process to select the training data set introduced a random component in model creation and evaluation. To minimize its impact, the sampling was repeated 100 times for each model, from which median and standard deviation of regression coefficients were extracted.

The estimation of the model skill was made on all data not comprised in the training set (also sampled 100 times). It should be noted that this validation set is imbalanced with respect to presences/absences, country representativeness, and datasets spatial overlap, thus it represents the hardest validation conditions (additional results for more balanced validation subsets are reported in Supplementary Information).

### 2.5 Model performance evaluation

To compare the modelled probability of occurrence with the reported absence-presence binary observations, a threshold of 50% probability was assumed, i.e. when modelled probability equaled or exceeded 0.50, the condition was considered present. The comparison gave four possible outcomes: fractions of true positives TP, true negatives TN, false positives FP, i.e. the model predicted occurrence of condition when WISE data reported absence, and false negatives FN, i.e. the model predicted absence of condition when WISE reported occurrence.

Models were evaluated with three metrics that are frequently used in classification problems and provide complimentary information on model performance:

(i) the overall accuracy (Acc), i.e. the fraction of cases that were correctly predicted (TP + TN). This metric is directly interpreted, but may be misleading when classes are imbalanced;

(ii) Cohen’s kappa coefficient (K_C_; Cohen, 1960, Ben-David, 2008). K_C_ compares model’s performance against a random classification. Theoretically K_C_ may range from 1 (perfect classification) to −1 (perfect misclassification), whereas 0 indicates the model performance is equal to a random classifier. There is no standardized way to interpret intermediate values; however, Landis and Koch (1977) suggested a scheme that is frequently taken as reference, whereby K_C_ < 0 indicates no agreement, 0–0.20 slight, 0.21–0.40 fair, 0.41–0.60 moderate, 0.61–0.80 substantial, and > 0.81 almost perfect agreement;

(iii) Area Under the Curve (AUC), i.e. the integral below the Receiver Operator Characteristic (ROC) curve. ROC curves are plots of model sensitivity (true positive rate, TP/(TP + FN)) versus specificity (true negative rate, TN/(TN + FP); Freeman and Moisen, 2008, Ben-David, 2008). The closer the AUC to 1, the higher TP associated to low FP, i.e. the better the model; vice versa, the closer the AUC to 0.5, the poorer the model.

All statistical analysis was conducted on R (R Core Team, 2014), being especially useful in the analyses the libraries MASS (Venables and Ripley, 2002) and pROC (Robin et al., 2011).

### 2.6 Mapping probability of occurrence at European scale

The European maps of probability were created applying the validated logistic regressions to the set of European indicators covering the EU-27, UK, NO, and CH, even though for the latter no information was available in WISE. Conversely, despite the United Kingdom fed data of ecological status and impacts into WISE-WFD dataset (Figure SI1), UK data could not be used to inform the logistic regressions as geographic information was missing (Table 1; Figure SI2). Thus, while UK and CH are included in the maps, results must be considered with caution as no data was used to validate them.

To limit the risk of extrapolation outside the range of explanatory variables applied in the training sets, the ranges of some WPIs were limited by setting maximum mean annual total nitrogen concentration (TN_mgL) at 1000 mg/L and maximum BOD concentration (BOD_mgL) at 5000 mg/L. In some coastal catchments, missing net abstractions were assumed nil and low flow alteration (Q10) was assumed unaltered compared to ‘natural’ conditions (Q10 = 0.11, equal to the median of the dataset). These adjustments and assumptions allowed covering the target region consistently, although predictions along coastal areas are to be taken as tentative. Maps and statistical distribution of the amended WPIs are shown in supporting information (Fig. SI4).

## 3 Results

Stepwise reduction resulted in models with five or six variables (Table 3); in all cases variance inflation factor was less than two, indicating low multi-collinearity in the final explanatory variable sets. The sampling procedure had little impact on the variability of the coefficients as indicated by the standard deviations of the 100 RSS repetitions.

**Table 3:** Coefficients of logistic regressions of probability of failing to achieve good ecological status (ES) and occurrence of nutrient pollution; organic pollution; chemical pollution; altered hydrology; altered morphology; and absence of impacts. The coefficients are the medians (in brackets standard deviation) of 100 random sampling repetitions. Since the explanatory variables were not standardized, the coefficients values do not reflect their importance for the outcome but depend on the range and the units of measure of the explanatory variables, as defined in Table 2. The most important explanatory variable according to AIC is highlighted in bold. AIC ranking of importance of all variables is reported in Supporting Information Table SI1.

	Failing good ES	Nutrient pollution	Organic pollution	Chemical pollution	Altered hydrology	Altered morphology	No impact
*_0_* – intercept	−0.985 (0.009)	−0.608 (0.050)	−1.240 (0.021)	0.073 (0.047)	−1.076 (0.058)	−0.456 (0.013)	0.462 (0.032)
β*_1_* Rain		−0.0007 (0.00006)		−0.0001 (0.00005)	0.0011 (0.00007)		
β*_2_* ArtiArea	5.629 (0.135)	1.560 (0.171)	2.005 (0.200)		1.110 (0.211)	**2.623** **(0.168)**	−6.093 (0.279)
β*_3_* AgriArea	**2.230** **(0.023)**					0.637 (0.026)	**−1.168** **(0.057)**
β*_4_* TN_mgL			−0.0031 (0.0010)				
β*_5_* NShareAgri		**2.210** **(0.052)**	**1.776** **(0.047)**				
β*_6_* NSharePoint	1.186 (0.075)	2.531 (0.111)	2.698 (0.115)				−0.741 (0.166)
β*_7_* UrbRunoffShare		0.872 (0.129)		1.543 (0.106)		0.592 (0.082)	
β*_8_* BOD_mgL						−0.021 (0.002)	
β*_9_* Q10				0.462 (0.124)	−0.830 (0.216)		
β*_10_* Abstraction	−0.003 (0.0005)		0.018 (0.001)	0.014 (0.001)	**0.029** **(0.002)**	0.011 (0.001)	0.016 (0.001)
β*_11_* NaturalFlood_RipFr	−0.048 (0.029)				0.884 (0.101)		
β*_12_* DamFreeLength		−0.326 (0.030)		**−0.932** **(0.045)**	−0.450 (0.052)		0.305 (0.043)

The median overall accuracy Acc of logistic regressions for the validation sets ranged from 0.55 to 0.73, K_C_ from 0.07 to 0.37, and AUC from 0.56 to 0.79 (Table 4). Overall, model performances could be judged from moderate (chemical pollution, altered hydrology and morphology) to good (ecological status, nutrient and organic pollution). In what follows we look more closely to some specific cases.

**Table 4:** Evaluation of the logistic regressions for predicting the probability of failing to achieve good ecological status (ES)or occurrence of nutrient pollution; organic pollution; chemical pollution; altered hydrology; altered morphology; and no impact. Results are reported for the validation dataset (i.e. all data except the training sample). Sample size, overall accuracy (Acc), Cohen’s kappa (K_C_), and Area under the Curve (AUC) are reported. The prevalence is the fraction of presences (ones) in the dataset and is reported for the entire abridged dataset (75801 entries, observed prevalence) and for validation set. Reported values are the medians of 100 repetitions of sampling procedure.

		Failing good ES	Nutrient pollution	Organic pollution	Chemical pollution	Altered hydrology	Altered morphology	No impact
*Observed prevalence*		*0.55*	*0.27*	*0.17*	*0.43*	*0.16*	*0.37*	*0.28*
Validation dataset	*Prevalence*	*0.58*	*0.23*	*0.15*	*0.43*	*0.15*	*0.34*	*0.25*
	sample size	51,221	63,181	68,591	64,565	71,117	62,717	66,567
	Acc	0.63	0.73	0.71	0.55	0.61	0.62	0.55
	K_C_	0.27	0.37	0.24	0.13	0.07	0.16	0.18
	AUC	0.68	0.79	0.76	0.56	0.58	0.60	0.69

### 3.1 Failing to achieve good ecological status

Model Acc of failing to achieve good ecological status was 0.63; K_C_ was 0.27, and AUC was 0.68. All the criteria indicate acceptable model performance. The accuracy of the logistic regression was slightly lower than the random forest model presented by Grizzetti et al. (2017), but the slight loss in accuracy is not significant and can be easily explained by the several differences in input data (area coverage and resolution), and methods (spatial overlapping, logistic regression vs machine learning approaches) between this and the previous study. The probability of failing to achieve good ecological status increased with increasing pressures from agriculture (fraction of agricultural areas in the catchment) or urbanized areas (fraction of artificial areas in the catchment and share of point sources to pollution load), while it decreased with increasing fraction of natural cover in riparian land and (unexpectedly) with abstractions (Table 3).

**Fig. 1:**
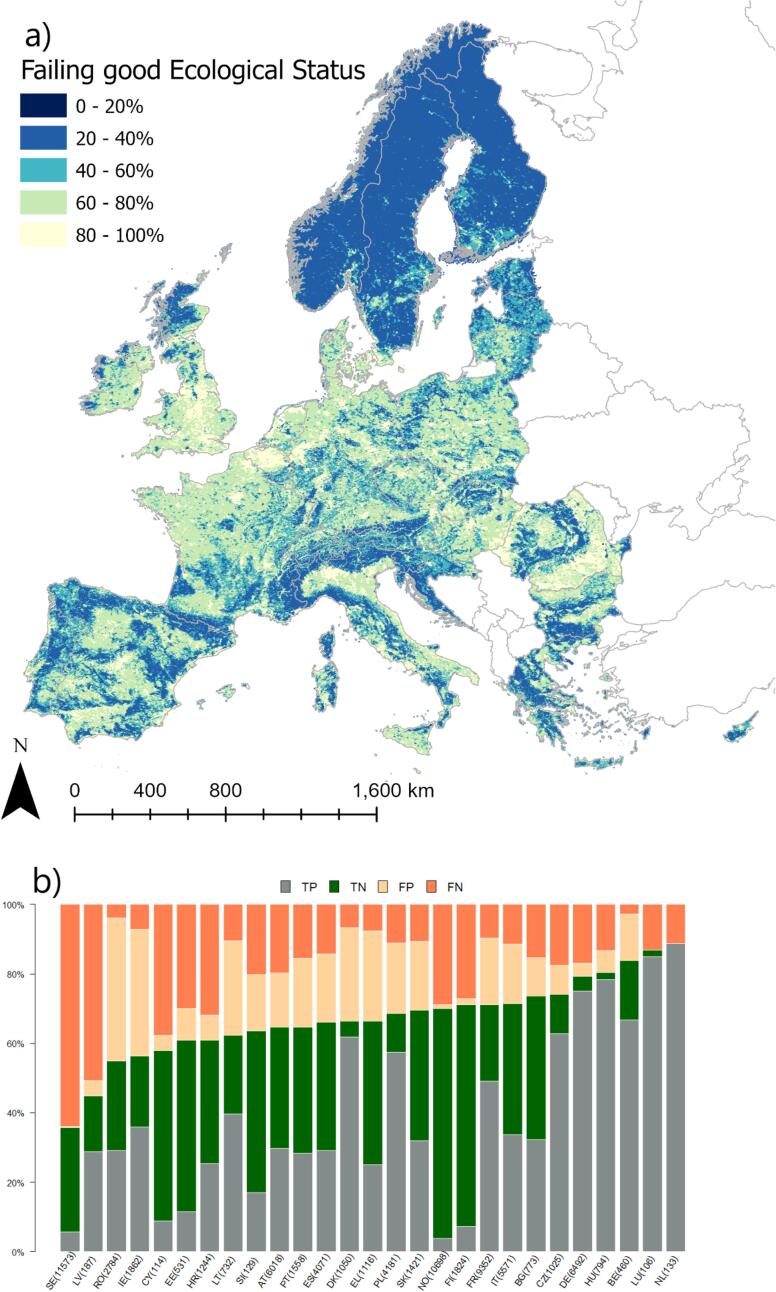
Modelled probability of failing to achieve good ecological status in rivers. (a) the European probability map; (b) distribution of true positive (TP), true negative (TN), false positive (FP, i.e. the model predicts failure to achieve good ecological status whereas the reported class does) and false negative (FN, i.e. the model predicts achieving good ecological status whereas the reported class does not) per country. Numbers in bracket indicate the number of rivers per country in validation dataset.

Looking at the distribution of discrepancies between model and reported status per country helps highlight where the model performed better (or worst; Fig. 1b). Most model errors consisted of false negative, i.e. the model indicated more rivers as achieving good ES than reported by countries. This is especially the case for SE and LV. Conversely, in RO and IE the model appears to underpredict occurrence of good ES, with a predominance of false positives.

### 3.2 Impact types

**Fig. 2:**
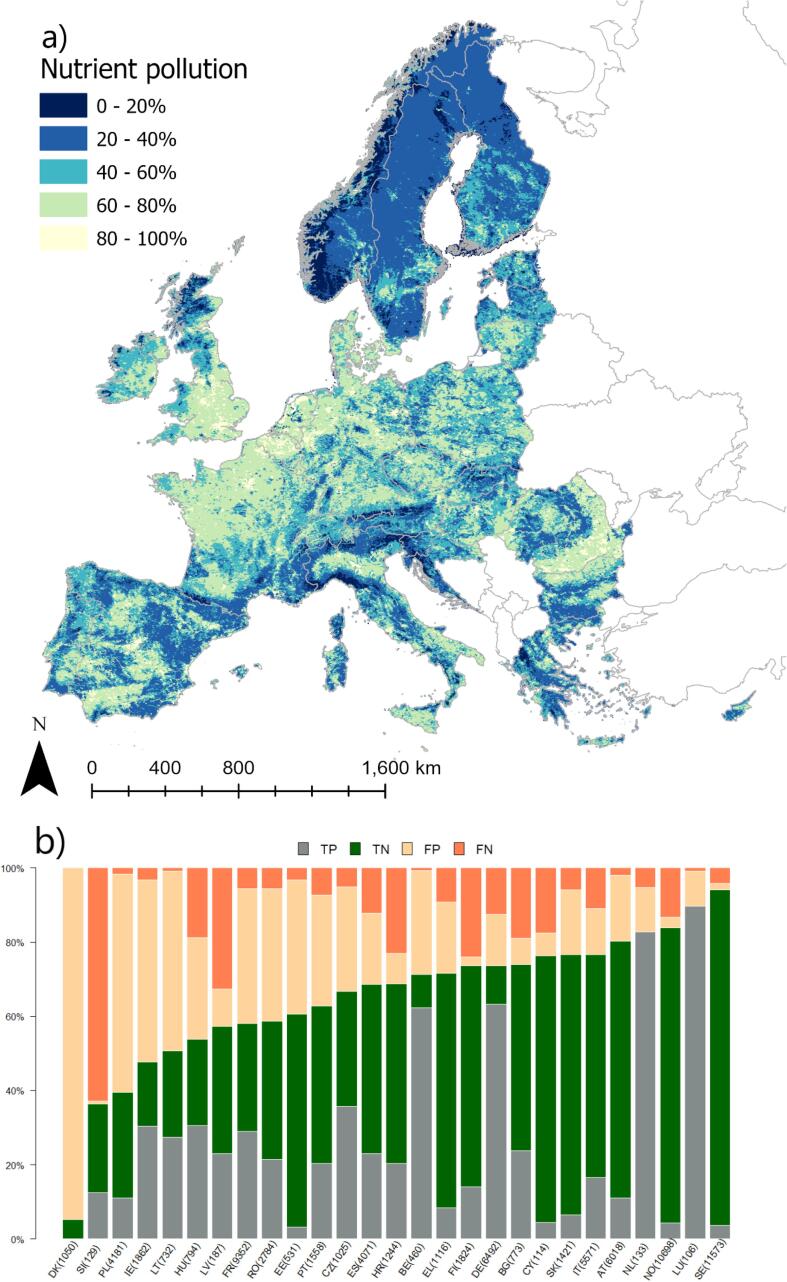
Modelled probability of occurrence of nutrient pollution in European rivers. (a) the European probability map; (b) distribution of true positive (TP), true negative (TN), false positive (FP) and false negative (FN) per country. Numbers in bracket indicate the number of rivers per country in validation dataset.

The success of the approach to predict occurrence of the most frequently reported impact types varied from good to moderate (Table 4). The best model results were obtained for nutrient pollution (Fig. 2a), whose validation even outperformed the probability of failing to achieve good ES. Occurrence of nutrient pollution was modelled to increase with the shares of nutrient loads from agriculture and point sources, the fraction of artificial land in the catchment, and the share of urban runoff. Conversely, rainfall gradient and dam-free length share had a negative (mitigating) role (Table 3). Maybe surprisingly, the mean annual nitrogen concentration in rivers was not retained in the final model. With hindsight, this can be explained by the fact that the variables retained in the regression are the drivers of nutrient pollution, whereas nitrogen concentration is the outcome of these sources, but attenuated by potential sinks and dilution effect of streamflow (Grizzetti et al., 2012).

The regression tended to overestimate the nutrient pollution occurrence compared to reported data, i.e. the number of FP was larger than FN (Fig. 2b). This was especially the case of DK, which did not report any nutrient pollution (Table 1), but for which the model indicated a pervasive nutrient pollution problem (Fig. 2a). Indeed, DK does not include consideration of nutrient concentrations in assessing ecological status (Poikane et al., 2019). On the other hand, in DK extensive adoption of nature based solutions of nutrient recycling and attenuation has been effective in curbing nutrient pollution (Kronvang et al., 2008). However, these management practices are not reflected in the explanatory variable set. Model FP may also be explained by ecological reasons, e.g. where phosphorous limits eutrophication, like in some regions in FR and IE, nutrient pollution impact is not reported notwithstanding potentially high nitrogen levels in the rivers.

**Fig. 3:**
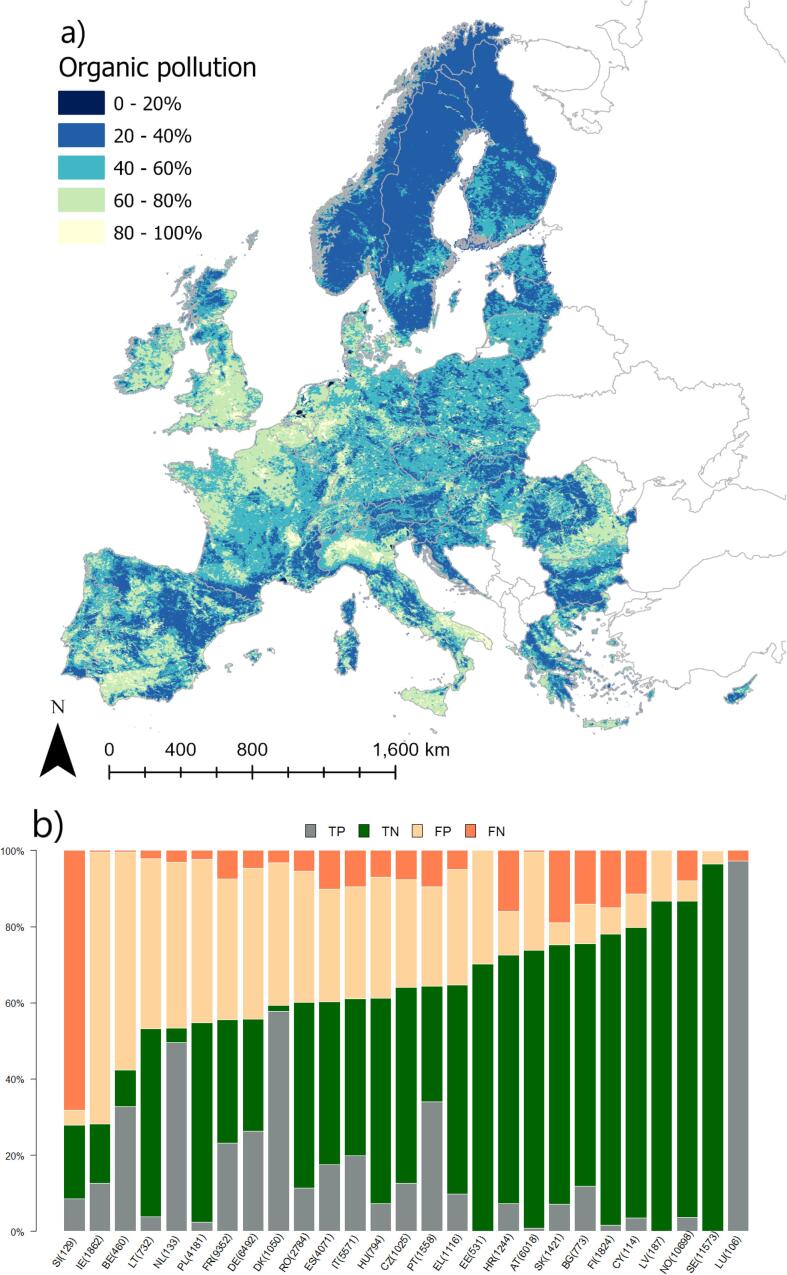
Modelled probability of occurrence of organic pollution in European rivers. (a) the European probability map; (b) distribution of true positive (TP), true negative (TN), false positive (FP) and false negative (FN) per country. Numbers in bracket indicate the number of rivers per country in validation dataset.

The organic pollution model was slightly less accurate (Table 4; Fig. 3) than the nutrient case; particularly K_C_ was low (0.24), due to a higher fraction of false positives than the nutrient pollution case. The organic pollution model shared almost the same main indicators as nutrient pollution, but with net abstractions rather than rainfall driving the hydrological gradient. Similarly to the case of nutrient pollution, BOD concentration was not included in the final regression, but again BOD concentration is the cumulative outcome of all processes regulating biochemical oxygen demand, including emissions, attenuation, uptake, and dilution of organic loads. Instead, the organic pollution model included nitrogen concentration variable with a negative gradient, whose interpretation is difficult.

**Fig. 4:**
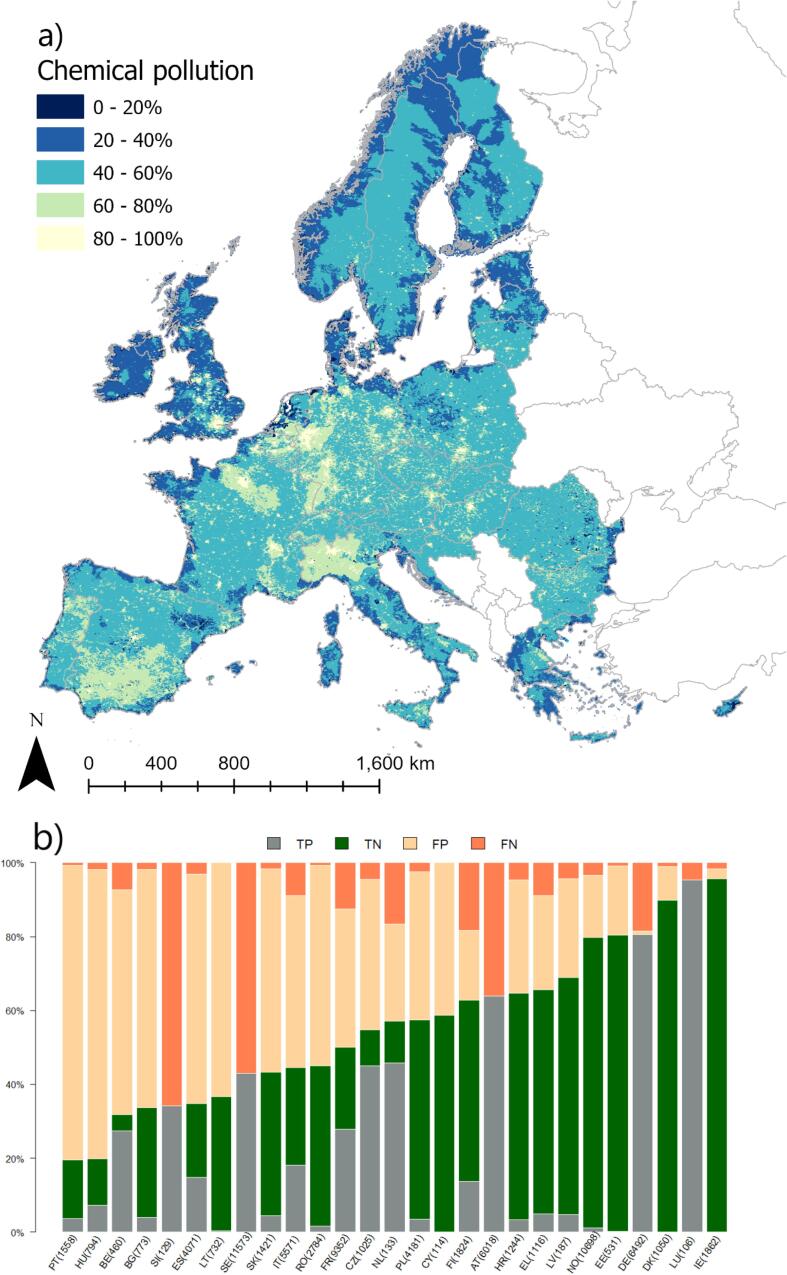
Modelled probability of occurrence of chemical pollution in European rivers. (a) the European probability map; (b) distribution of true positive (TP), true negative (TN), false positive (FP) and false negative (FN) per country. Numbers in bracket indicate the number of rivers in validation dataset.

Conversely, the chemical (i.e. excluding nutrient and organic pollutants) pollution model was the worst (Fig. 4). Despite a large training sample size and a rather balanced observed prevalence (43%), model performance dropped considerably from training to the validation, resulting in Acc of 0.55, K_C_ of 0.13, and AUC of 0.56 (Table 4). Acc raised over 50% only in 15 out of the 27 countries (Fig. 4b). Therefore, while the results are presented here for completeness, the map (Fig. 4a) is not to be used for practical purposes. A clear country reporting effect can be detected in this case (Fig. 4b). Some countries considered the presence of ubiquitous substances, such as for example mercury, in their assessment of chemical status (and pollution; EEA, 2019). Other countries elected to discard consideration of these substances in the evaluation of chemical status and pollution (EEA, 2019). This heterogeneity in reporting choices hampers assessment of gradient patterns of chemical pollution and its drivers. For example, in considering ubiquitous substances, SE, AT, and SI reported all river water bodies being impacted by chemical pollution (Table 1). According to the regression, chemical pollution in these countries is sizably lower (large shares of false negatives). Had all countries taken the same choice, the probability of occurrence of chemical pollution would be 1 everywhere.

The main explanatory variable for chemical pollution appears to be in fact a “mitigating” variable, i.e. DamFreeLength (Table 3), suggesting that the model captures better situations not affected by pollution (which may tend to occur in less disturbed parts of the stream network). Chemical pollution is modelled as increasing with urban runoff share, abstractions and alteration of low flow (Q10), suggesting that high use of water might be coupled to higher chemical emissions. Finally, rainfall appears to have a mitigating effect, possibly connected to dilution. Despite the fact that the explanatory variables that emerge from the analysis are logically and physically meaningful, the inconsistency in reported impacts among countries is likely to play such a role as to undermine the applicability of a statistical model in this case. In general, the variability in number and pathways of chemicals of concern makes any assessment of chemical pollution impact very difficult, and the knowledge of contaminants from modelling or monitoring remains insufficient to characterize chemical pollution (Posthuma et al., 2020). Nevertheless, recent efforts to build consistent continental scale picture of chemical pollution (Pistocchi et al., 2019, Van Gils et al., 2020) may advance detection of chemical pollution impact patterns (Lemm et al., 2021).

**Fig. 5:**
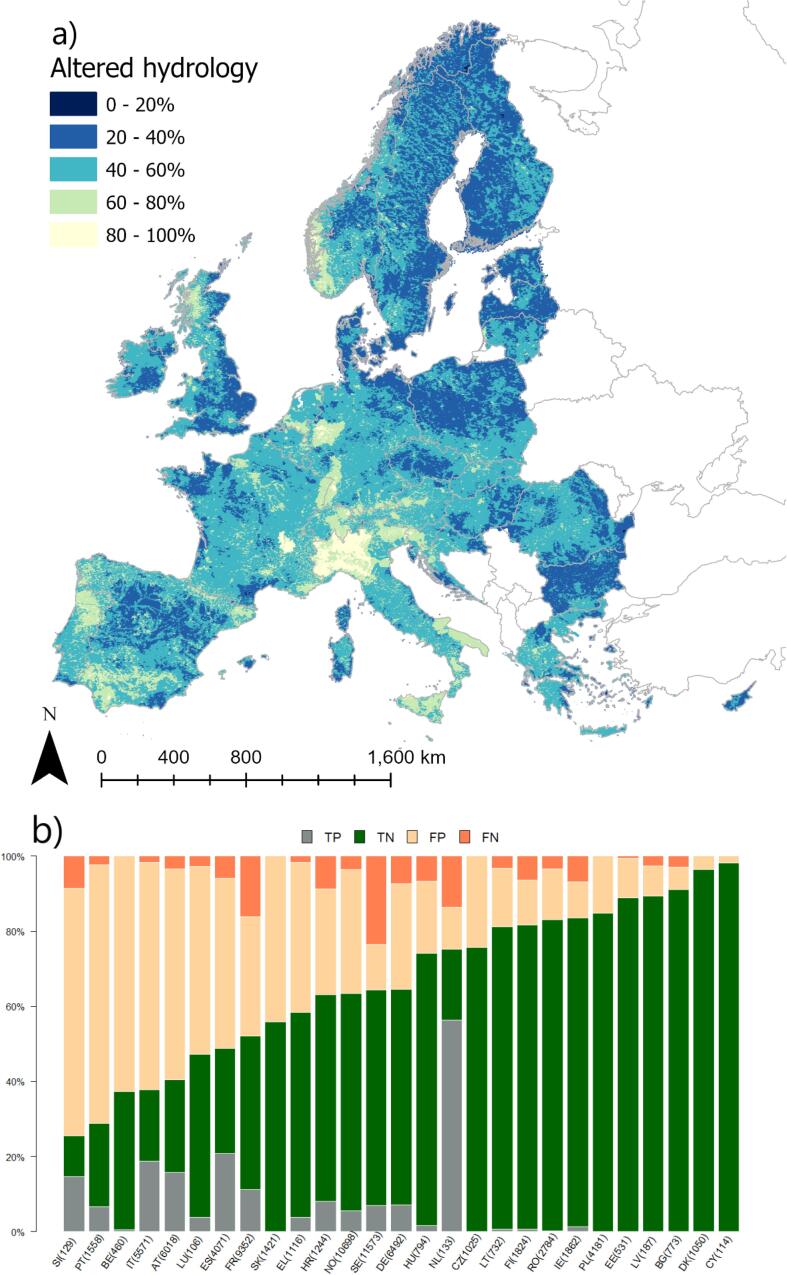
Modelled probability of occurrence of hydrological alteration of habitats in European rivers. (a) the European probability map; (b) distribution of true positive (TP), true negative (TN), false positive (FP) and false negative (FN) per country. Numbers in bracket indicate the number of rivers in validation dataset.

Occurrence of hydromorphological alterations of habitats was difficult to predict. Acc (0.61) and AUC (0.58) of occurrence of hydrological alteration of habitats were similar to morphological alterations (Acc = 0.62, and AUC = 0.60; Table 4). Conversely, K_C_ was remarkably lower for hydrological alterations (0.07) than for morphological alterations (0.16). The final model of hydrological alteration occurrence comprised six indicators, with an increase of probability attributed to abstractions, rainfall, share of artificial areas, and (unexpectedly) the fraction of natural land cover in riparian areas and floodplains. Actually the probability pattern appears dominated by rainfall and abstractions (Fig. 5a). A mitigating influence was instead associated with dam free length share. The low flow alteration index (Q10) was the sixth variable in order of importance in the regression, however the coefficient sign was, counterintuitively, negative. Thus, alteration of low flow was not captured in the model as such; rather, the model was dominated by the pattern of abstractions and partly counteracted by the DamFreeLength share, which was the 4th indicator per importance, with a negative sign (Ranking of coefficients is reported in SI, Table SI1). The model tended to overestimate hydrological alteration, with a predominance of false positive (Fig. 5b) everywhere but in SE.

**Fig. 6:**
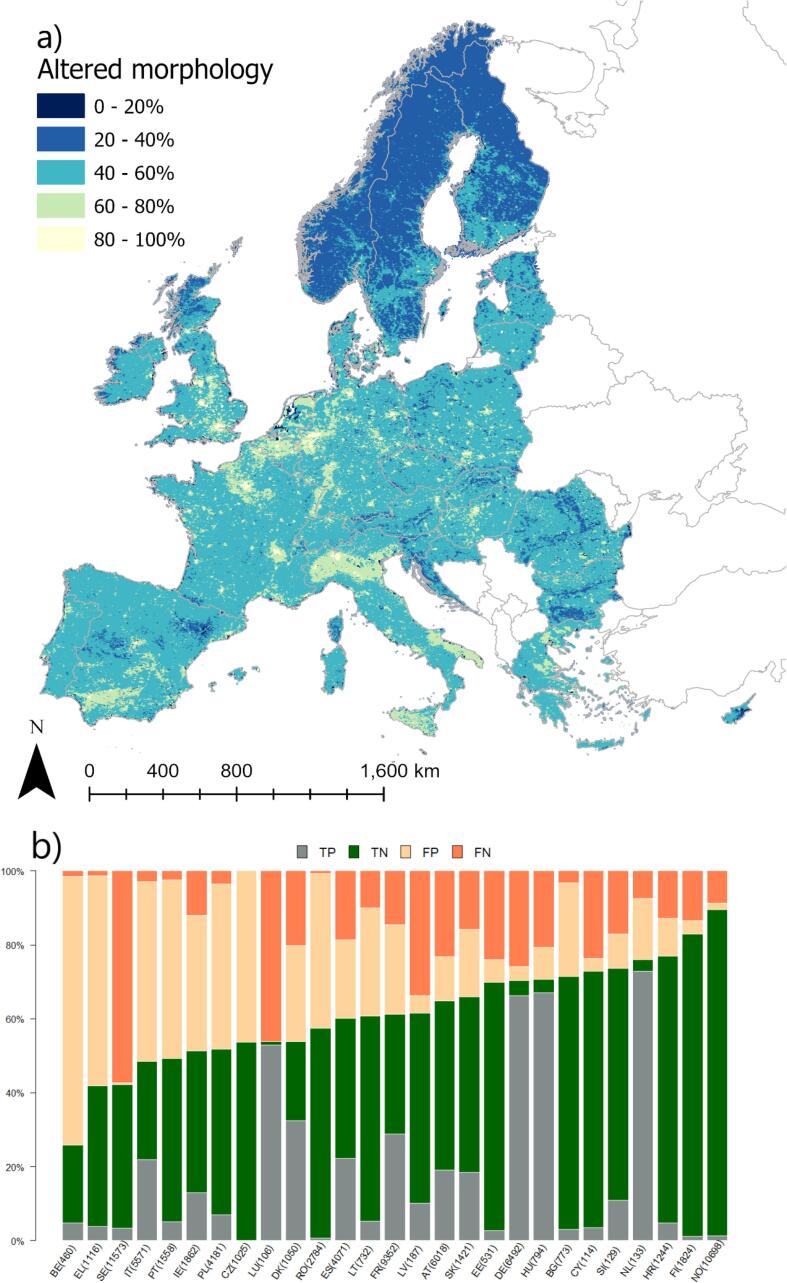
Modelled probability of occurrence of morphological alteration of habitats in European rivers. (a) the European probability map; (b) distribution of true positive (TP), true negative (TN), false positive (FP) and false negative (FN) per country. Numbers in bracket indicate the number of rivers in validation dataset.

Despite scoring slightly better in terms of validation performance, the model for morphological alteration occurrence was equally unsatisfactory; an intermediate morphological alteration was predicted to be quite pervasive over the continent (Fig. 6a), which is somehow expected as Europe shows a relatively uniform and high population density. Increasing probability of morphological alteration was related mainly to the presence of artificial and agricultural land cover in the catchment, followed by abstractions and urban runoff share, whereas a negative gradient was associated to BOD concentration.

**Fig. 7:**
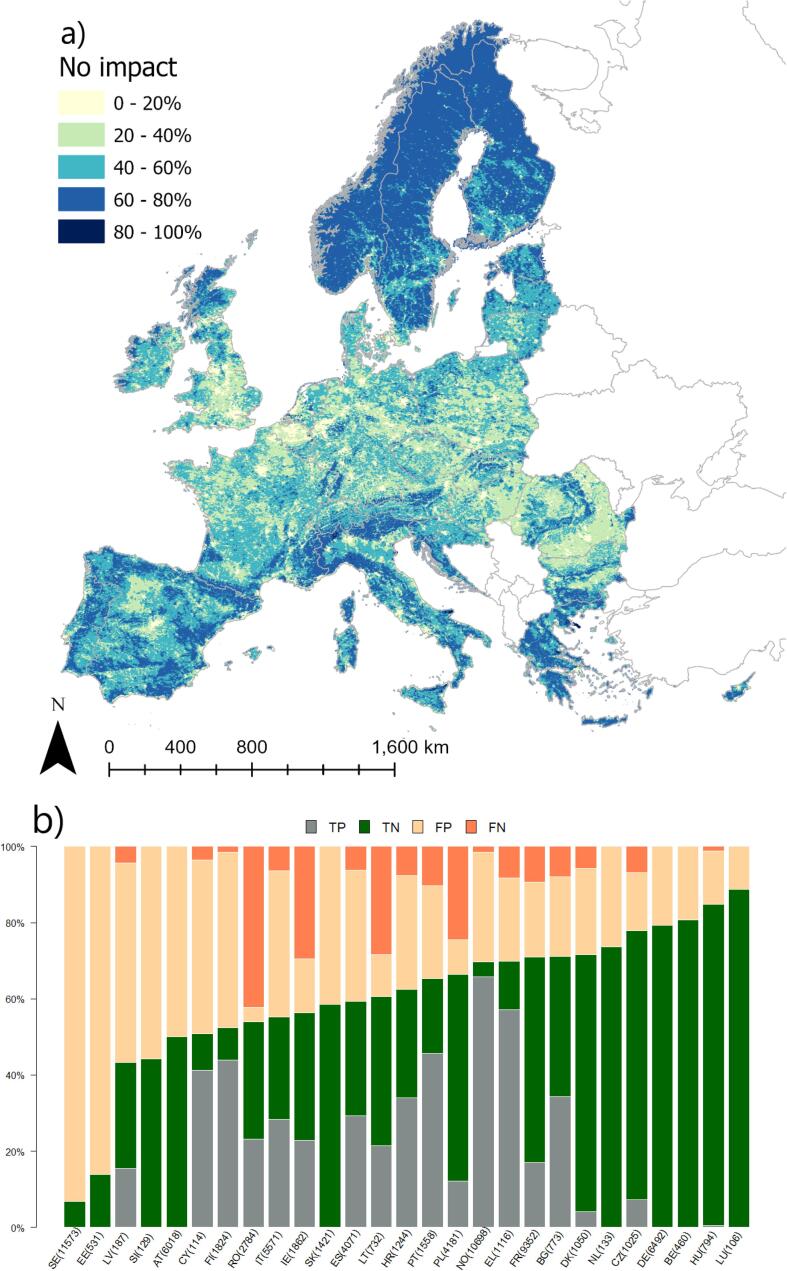
Modelled probability of occurrence of no impact in European rivers. (a) the European probability map; (b) distribution of true positive (TP), true negative (TN), false positive (FP) and false negative (FN) per country. Numbers in bracket indicate the number of rivers in validation dataset.

The pattern of no impact mirrored that of failing to achieve good ES, although extreme probabilities were less frequent (Fig. 7a). The performance of logistic regression predicting no impact was intermediate; AUC was similar to that of failing to achieve good ES; but Acc and K_c_ were lower. The model variables indicated that the higher the human presence in terms of share of artificial and agricultural land, and nutrient pollution from point sources, the higher the occurrence of some impacts. Dam free length share and (unexpectedly) abstractions were found to increase probability of no impact. The number of false positive was the major error type, and comprised 37% of all cases. These results suggest that something in explaining the absence of impacts is amiss. The absence of impacts implies by definition that no other impact has been reported, so for example declaring presence of chemical impact everywhere may explain part of the failures (e.g. in SE, AT, SI, LU; Fig. 7b). Furthermore, the presence of ‘unknown’ impacts (Table 1) may explain another important share of failures, e.g. in EE. Identifying these unknown impacts will thus also help better locating rivers with no impact.

## 4 Discussion

### 4.1 Statistical method

Model predictions are affected by sampling method, whereby balanced sampling reduces uncertainty and improve precision of parameter estimations (Salas-Eljatib et al., 2018). Our choice of using balanced samples reduced the potential sample size, however training samples were always large enough (>4000, Table SI3) to avoid small sample inference biases. Balanced samples may affect model predictions, potentially leading to reduced sensitivity or specificity when prevalence of the sampled population is imbalanced. In our case, the observed prevalence in the abridged dataset (Table 4) alone does not explain validation results. For example, the prevalence of organic pollution and altered habitat due to hydrological changes is about 16%, but model predictions in the first case are much better than in the second. Additionally, chemical pollution prevalence is 43%, yet model performance was poor. Balanced conditions are important also for validating models: indeed, using a balanced validation subset largely confirmed model training and testing (see supporting information). Finally, repeating the sampling 100 times demonstrated that variability of coefficients due to random sampling was small (Table 3, or coefficient ranges in Table SI1). We can thus conclude that sampling procedure did not introduce unwarranted bias.

Acc and K_C_ are sensitive to the threshold used to separate positive and negative cases, especially if prevalence is far from 50% and if the model is poor (Freeman and Moisen, 2008, Ben-David, 2008). In this study, the presence-absence threshold was set at 0.50, which is the simplest and often the default choice. However, other criteria may be equally valid (Freeman and Moisen, 2008). Fluet-Chouinard et al. (2020) argued that for reported presence/absence data types, thresholds could be adjusted to better reflect perceptions of pressures and links with habitat condition. The probability thresholds that best matches binary information and maximize K_C_ (i.e. minimize errors) can be identified from the ROC curves (Fluet-Chouinard et al., 2020), and equaled to 0.52 for failing to achieve ES and no impact, 0.42–0.43 for nutrient, organic and chemical pollution, 0.51 for hydrological alteration and 0.46 for morphological alteration model (Table SI2). The choice of a 50% threshold was thus quite insensitive for the analysis. While each performance criterion suffers shortcomings, the interpretation of all criteria together (and ROC curves, in SI) points to good performance for failing to achieve good ES, nutrient and organic pollution, moderate for no impact, and poor for the other cases.

### 4.2 Sources on uncertainty

**Fig. 8:**
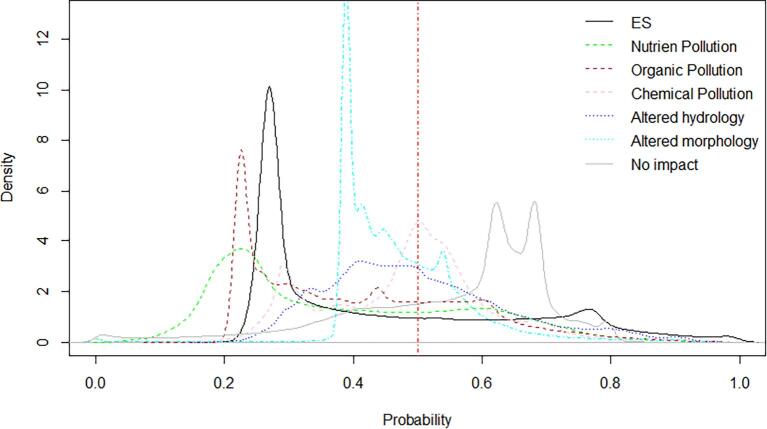
Density plots of probability of occurrence depicted in the probability maps. The red line shows the 50% threshold used to separate absences and presences in map evaluation. ES: failing to achieve good ecological status. (For interpretation of the references to colour in this figure legend, the reader is referred to the web version of this article.)

**Fig. 9:**
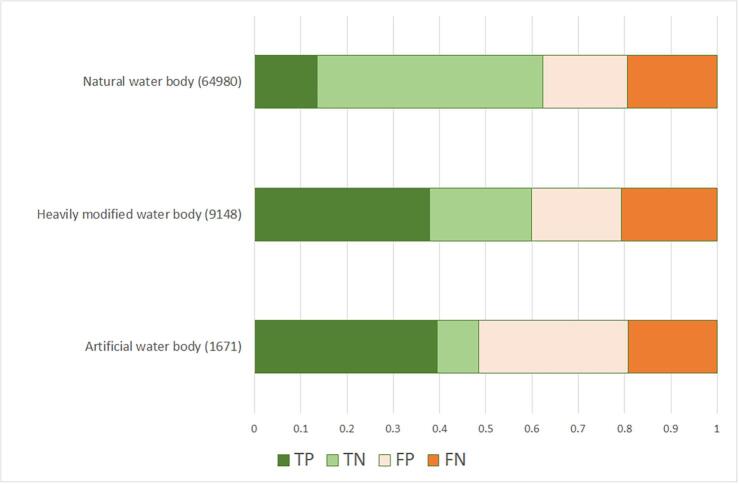
Results of modelled probability of occurrence of altered morphology by river category. TP = true positive, TN = true negative, FP = false positive FN = false negative. Number in brackets indicate data size.

Several sources of bias and uncertainty can explain model performances. Fig. 2, Fig. 3, Fig. 4, Fig. 5, Fig. 6, Fig. 7, Fig. 8, Fig. 9 show that choices of countries in reporting impacts (Table 1) have an important influence on results; this effect is detectable in all models, but especially for chemical pollution. Fluet-Chouinard et al. (2020) note that epistemic uncertainty may affect national reporting presence of pressures: besides monitoring data basis, perceived links between impacts and ecological status as well as frequency of occurrence of an impact may affect it. Intercalibration efforts (Poikane et al., 2014) supported more consistent reporting of ecological status among countries. Furthermore, they strengthened the cause-effect links between ecological responses and anthropogenic pressures, favoring the use of biological quality indicators that are diagnostic for pressures causing failures to achieve good ES (Poikane et al., 2020). This is improving the knowledge basis of ecological status and nutrient pollution impact, where major efforts are undergoing in critically defining nutrient thresholds (Poikane et al., 2020). Not by chance, the probability models for failing good ES and nutrient pollution are the best found in this study. Conversely, monitoring and reporting hydromorphological alterations is still heterogeneous, also due to the variety of issues covered under this topic (Kampa and Bussettini, 2018, Santos et al., 2021). Extending intercalibration efforts to better define links between hydro-morphological alterations or chemical pollution on ecological status will likely improve reporting of these impacts as well.

In turn, the explanatory variables used to build the regressions are affected by errors due to incomplete or erroneous input data, which may generate heterogeneous biases. This could be for example the case of loads from point sources, as mapping of domestic and industrial discharges is still uneven and incomplete (Pistocchi et al., 2019, Vigiak et al., 2020). Additionally, variables derived from models, like pollutant concentrations or flow regime alteration, are also affected by structural uncertainty of models adopted (Grizzetti et al., 2012, Vigiak et al., 2018, Vigiak et al., 2019).

The most important indicators explaining river impacts were share of artificial land, share of urban runoff in flow, pollution loads from agriculture and point sources, mean annual net abstractions, and the share of upstream river length uninterrupted by barriers (Table 3). For the most part, the signs of regression coefficients confirmed intuition about potential direct and indirect effects of water pressure indicators on river conditions, but with some exceptions. For example, the share of natural areas in floodplains was found to increase the probability of achieving good ecological status, which is in accordance with Grizzetti et al. (2017) and with Lemm et al. (2021, who however used the shares of artificial and agricultural in riparian land). On the other hand, it was associated with an increased probability of occurrence of altered hydrology. This is counterintuitive given the multiple benefits associated to natural vegetation in riparian land (Cole et al., 2020, Park and Lee, 2020), and partly in contradiction with Schmidt et al. (2019), who found that natural land in floodplains was beneficial to river habitats. A possible explanation may lay in the resolution of spatial units and the ensuing range of shares of riparian land. In this study, the use of small catchments resulted in very skewed distribution of the variable; with the majority of values at zero and a small fraction of values a 100% with very few values in between (Fig. SI4k). Park and Lee (2020) observed that the effect of land use is not spatially stationary and may become detectable above some threshold fractions. Furthermore, the small size of CCM2 catchment may have also be the cause of errors when resampling abstractions and low flow alteration variables, originally provided in 5 × 5 km^2^ grids (Bisselink et al., 2018).

The spatial intersection of geometric features from the two datasets introduces further uncertainty. The selection for model training of pairs whose geographic features overlap for at least 50% increased in theory the chances that CCM2 catchment water pressure indicators were representative of the WISE-WFD river water body. However, it is interesting to note that the extent of spatial overlap between geodatasets did not affect validation results: performance of a balanced validation subset extracted from pairs with overlap <50% was as good as for training and testing (Table SI3), therefore *a posteriori* we can conclude that separating the two groups had no impact on the logistic regressions, or that the uncertainty introduced by spatial intersection was overall minor.

### 4.3 Knowledge gaps

While all the above uncertainties and potential errors must be acknowledged, they cannot explain the causes of failing to detect occurrence of some impacts entirely. Fig. 8 shows the distribution density of the probability maps. It is interesting to note that failing good ES, nutrient and organic pollution have two distinct modal peaks: a major one around 20% of probability and a second one at around 60%. Chemical pollution shows two peaks, one around 30% and a main one at 50%, but most predictions concentrate in the rather small interval of 30–60%. Likely, the peak around 30% is inflated by assumptions of abstractions and low flow alteration taken in the coastal areas (section 2.6; Fig. 4a). Conversely, altered habitats by hydrology has one single peak at around 40%, whereas altered habitats due to morphology starts at probability around 38%, has a main peak at 40% and second one at 55%.

The probability region from 40 to 60% is a sort of ‘grey zone’: a slight change in one explanatory variable may shift an assessment toward absence or presence of a condition. The presence of two clear peaks indicates that the explanatory variable set can easily identify regions of low or high probability, whereas a high number of cases in the 40–60% region or a small range of probabilities suggest that the model is likely missing some key information to clearly identify the presence of the condition. This appear to be the case for altered hydrology. It is worth considering here that altered hydrology may be due to very different processes, like hydropeaking, flood control, over-abstractions. Under such generic umbrella, it may be difficult to disentangle pressures and management issues. Maybe subdividing altered hydrology types might help in the future to better select relevant water pressure indicators. At the same time, these alterations are also reflected by different properties of flow regimes (magnitude, frequency, duration, etc; Kampa and Bussettini, 2018); enlarging the explanatory variable sets to include other flow regime indicators in addition to low flow alteration, particularly choosing among those more robust to detect regime alteration (Vigiak et al., 2018), could be beneficial to the analysis.

Similar considerations can apply to the occurrence of morphology alteration case. The main explanatory variable directly referring to morphology alteration is the Dam Free Length Share, which focuses on longitudinal fragmentation. While pivotal, this is only one aspect of morphological alteration, that is much larger and encompasses lateral connectivity, bed substrate, channel adjustments, cross section configuration, etc (e.g. Golfieri et al., 2018, Kampa and Bussettini, 2018). Furthermore, the indicator Dam Free Length Share (Pistocchi et al., 2018), which accounts only for the large dams, is likely underestimating the actual disruption of longitudinal connectivity. A very recent study (Belletti et al., 2020) showed that river fragmentation in Europe is much more pervasive than previously assessed. While large dams are particularly important for altered hydrology as identified also in our study (Table 3), small barriers and river structures constructed for flood control, navigation, or stabilization of river beds have large impacts on morphological alteration (Belletti et al., 2020). If we look at distribution of false predictions per river category (Fig. 9), we can see that model accuracy drops from natural rivers to heavily modified and to artificial water bodies. Engineered rivers are clearly impacted by altered morphology, but no suitable explanatory variable could be included in the initial set (Table 2). Hopefully, the newest inventory of European barriers (Belletti et al., 2020) may improve predictions of morphological alteration; this issue should be further pursued in future research.

This example points to a scale issue too, which is particularly important in the altered morphology case (Villeneuve et al., 2018, Golfieri et al., 2018). Most of the explanatory variables (Table 2) are catchment-scale indicators, i.e. they integrate over all upstream area conditions. Artificial and agricultural areas, as well as the fraction of natural vegetation in riparian land are the only indicators that are defined at the scale of the reach, or more precisely at the scale of the CCM2 catchment hosting the reach. However, both urban and agricultural land in a catchment are significantly correlated to the extent of the same land use type in their upstream areas, so it is hard to judge if these indicators can be considered of local or catchment scale (Park and Lee, 2020). This leaves the natural vegetation as the only reach scale factor included in the analysis, and can be considered a proxy for morphological conditions, reflecting in part vegetation along river banks (Lemm et al., 2021). However, no explanatory variable relates to riverbed, river banks, or substrates, which instead were found to have sizable effects on hydromorphological alteration of streams (Villeneuve et al., 2018).

### 4.4 Patterns of river conditions

Given the results summarized in Table 3 and the limits highlighted herein, the probability maps of occurrence of failing to achieve good ecological status, nutrient and organic pollution are considered robust, and offer a dependable snapshot of river European conditions. These maps offer a consistent visualization of river conditions at continental scale, and provide a more nuanced information compared to binary (presence-absence) reported data. They could be used for example to fill-in potentially knowledge gaps in reported data, or further progressing intercalibration efforts. Conversely, the several shortcomings of the current water pressure indicators to assess chemical pollution, hydro-morphological alterations, or no impact indicate the need for further research. As a consequence, these maps are not disseminated publicly nor discussed herein.

The probability of failing to achieve good ecological status was estimated to be >60% in 36% of river length of the 1,6 M km of river network comprised in Fig. 1a. These rivers are mostly located in the heavily urbanized and industrialized floodplains of the continent. The pattern of Fig. 1a confirms and expands that of Grizzetti et al. (2017), now including CH, EL, CY, all IT, and NO, and provides an estimate of river conditions for the 5% of river water bodies whose ecological status is reported as ‘Unknown’. The inclusion of EL and CY significantly improve coverage of probability mapping in the Mediterranean region, increasing confidence in estimations of river conditions in areas for which ecological status is not reported but for which water pressure indicators are available, e.g. covering MT, south of IT, and potentially extendible to all the Balkans.

Nutrient and organic pollution maps further shed light on the pressure-impact relationships. For example, the model of Nutrient Pollution puts emphasis on the direct drivers of pollution, i.e. share of nitrogen loads from agriculture and points sources, and share of urban runoff (Table 3), thus highlighting the impact of pollution on downstream reaches. The continental snapshot of nutrient pollution (Fig. 2a) results smoother than for ecological status, and indicates that occurrence of nutrient pollution at probability >60% extends over 26% of the river network length mapped area. Organic pollution occurrence is less diffuse, with a probability > 60% estimated for 20% of the river network.

### 4.5 Utility of results for water management.

The continental overview provided by the probability maps is very useful for policy making at European scale; the maps highlight the extent of nutrient and organic pollution, identify hotspots, and link visually pollution to the WFD ecological status. The impact type maps (Fig. 2, Fig. 3) could also be used for further analysis, e.g. in developing cumulative stress indices (e.g. Fluet-Chouinard et al., 2020).

Logistic regressions can further help inform decision-making process when exploring ‘what-if’ scenarios dealing with complex issues, such as for example when considering multiple impacts of urbanization (e.g. Salerno et al., 2018). The results of this study (Table 3) show how expansion of shares of urban and agricultural land, directly (β_2_ and β_3_) or through increases of associated pollution loads (β_5_, β_6_ and β_7_), may impact rivers in different ways. The importance of land use as major explaining variable affecting aquatic ecology is in line with other studies (e.g. Villeneuve et al., 2018, Glendell et al., 2019, Schmidt et al., 2019, Tang et al., 2020). However, interpretation of regression coefficients must be taken cautiously as logistic regressions, as other data-driven empirical models, do not identify cause-effect relationships. Related to this, it should be noted that the marginal effects of logistic regressions (Table 3) do not quantify the impact of each explanatory variable per se, but are the expression of all direct and indirect effects of the variable in the context of the whole variable set. Structural equation models built on cause-effect hypothesis (e.g. Villeneuve et al., 2018, Schmidt et al., 2019, Tang et al., 2020) would be more robust for disentangling cause-effect relationships. Nevertheless, in the light of the validation results, the logistic regressions developed for failing to achieve good ecological status and occurrence of nutrient and organic pollution could be used for such type of analysis.

The results of this study are of interest at country scale too. Since the maps build on knowledge gathered at continental scale, they are less influenced by the single country perspectives. Clearly, the maps suffer shortcomings due to lack of local data and errors. Nevertheless, countries could compare their own assessment with results published herein, and evaluate to which degree errors in predictions (FP and FN of Fig. 1, Fig. 2, Fig. 3, Fig. 4, Fig. 5, Fig. 6, Fig. 7, Fig. 8, Fig. 9) reflect lack of local knowledge of the models or potential underestimation or overestimation of freshwater river conditions. This assessment can be extended to all impacts analyzed in this study, and we believe this could reinforce intercalibration exercises within the WFD efforts, and in turn improve understanding of river conditions and pressure-impacts relationships.

Last but not the least, these results could be useful for basin water managers in evaluating potential river impacts for which no other information may be locally available. The results of this study cannot replace local analysis, but could signal the presence of some impacts that had not been previously considered, or for which no knowledge is available. In these cases, the maps provided may offer an initial estimate to consider basin management actions. This for example could be interesting in the cases of organic pollution or absence of impacts. Conversely, where local knowledge is essential, e.g. in the altered morphology case, the use of this study results at basin scale is not recommended.

## 5 Conclusions

This study developed European probability maps for occurrence of failing to achieve good ecological status and occurrence of most frequent impact types on rivers. The probability maps provide homogeneous, continental-scale snapshots of river conditions that blend information reported by countries through legislative channels with continental-scale water pressure indicators developed on independent models and data.

These findings update and expand previous work on ecological status (Grizzetti et al., 2017), confirming the relevance of European Water Pressure Indicators to assess freshwater conditions. The analysis on major impacts highlighted that assessing occurrence of nutrient and organic pollution was accurate and reliable, whereas detecting other impacts proved more difficult. Intercalibration efforts in reporting condition in the second RBMPs round has already advanced knowledge of ecological status and nutrient pollution; similar work in the future should also include hydro-morphological pressures and (other) chemical pollution. Concurrently, research should address developing pan-European pressure indicators suitable to characterize patterns of chemical pollution and hydromorphological alterations of habitats, as well as less frequently reported impacts, like acidification.

The probability maps can be used to assess the importance of major impacts on river pressures affecting ecological status. They highlight priority areas of intervention, and may fill in knowledge gaps in data reported through legislative channels. This applies for the probability of ecological status, nutrient and organic pollution, but is not recommended for other impacts for which the relationships appear too weak. The logistic regressions on which the probability maps are built could be used to explore ‘what-if’ scenarios to guide management affecting the main drivers of any given impact. This could be useful for example to assess multiple impacts due to the same driver, for example the effect of urbanization. However, it is important to remember that data-driven regressions do not identify cause-effect relationships, but are dependent on all conditions coexisting in the datasets, thus scenario results should be treated with caution.

Data availability

Probability maps of failing to achieve good ecological status, and occurrence of nutrient and organic pollution can be accessed at the JRC data catalogue (https://data.jrc.ec.europa.eu/collection/wpi) available from June 2021, and upon request to corresponding author before then.

## CRediT authorship contribution statement

**Olga Vigiak:** Conceptualization, Methodology, Formal analysis, Investigation, Data curation, Writing - original draft. **Angel Udias:** Methodology, Formal analysis, Investigation, Writing - review & editing. **Alberto Pistocchi:** Conceptualization, Writing - review & editing. **Michela Zanni:** Data curation, Visualization. **Alberto Aloe:** Data curation, Visualization. **Bruna Grizzetti:** Supervision, Conceptualization, Methodology, Investigation, Writing - review & editing.

## Declaration of Competing Interest

The authors declare that they have no known competing financial interests or personal relationships that could have appeared to influence the work reported in this paper.
