# Supplementary material for: Probability maps of anthropogenic impacts affecting ecological status in European rivers
Source: Ecol Indic. 2021 Jul;126:107684. doi: 10.1016/j.ecolind.2021.107684 (PMC8098054; doi:10.1016/j.ecolind.2021.107684)
Supplement: Supplementary data 1 [file mmc1.docx]

Supporting information

Index

1) Preparation of data for analysis, including (i) initial appraisal of WISE-WFD database; (ii) spatial overlapping and definition of abridged WISE-CCM2 pair dataset population and grouping by overlapping fractions ( ‘wb>50’ and ‘wb<50’ groups), and (iii) checking representativeness of groups.

2) Maps of WPIS used to derive probability maps + density plots of wb>50% group for training, wb<50 group for validation, and in maps

3) Pearson’s correlation of Water Pressure indicators in the wb>50 group used to extract training datasets

4) Table SI1: range and ranking of logistic regression coefficients

5) Probability map errors vs ecological status

6) Logistic regression ROC curves and optimal K_C_ threshold

7) Validation of models on balanced subsets

1) Preparation of data for analysis, including (i) initial appraisal of WISE-WFD database; (ii) spatial overlapping and definition of abridged WISE-CCM2 pair dataset population and grouping by overlapping fractions ( ‘wb>50’ and ‘wb<50’ groups), and (iii) checking representativeness of groups.


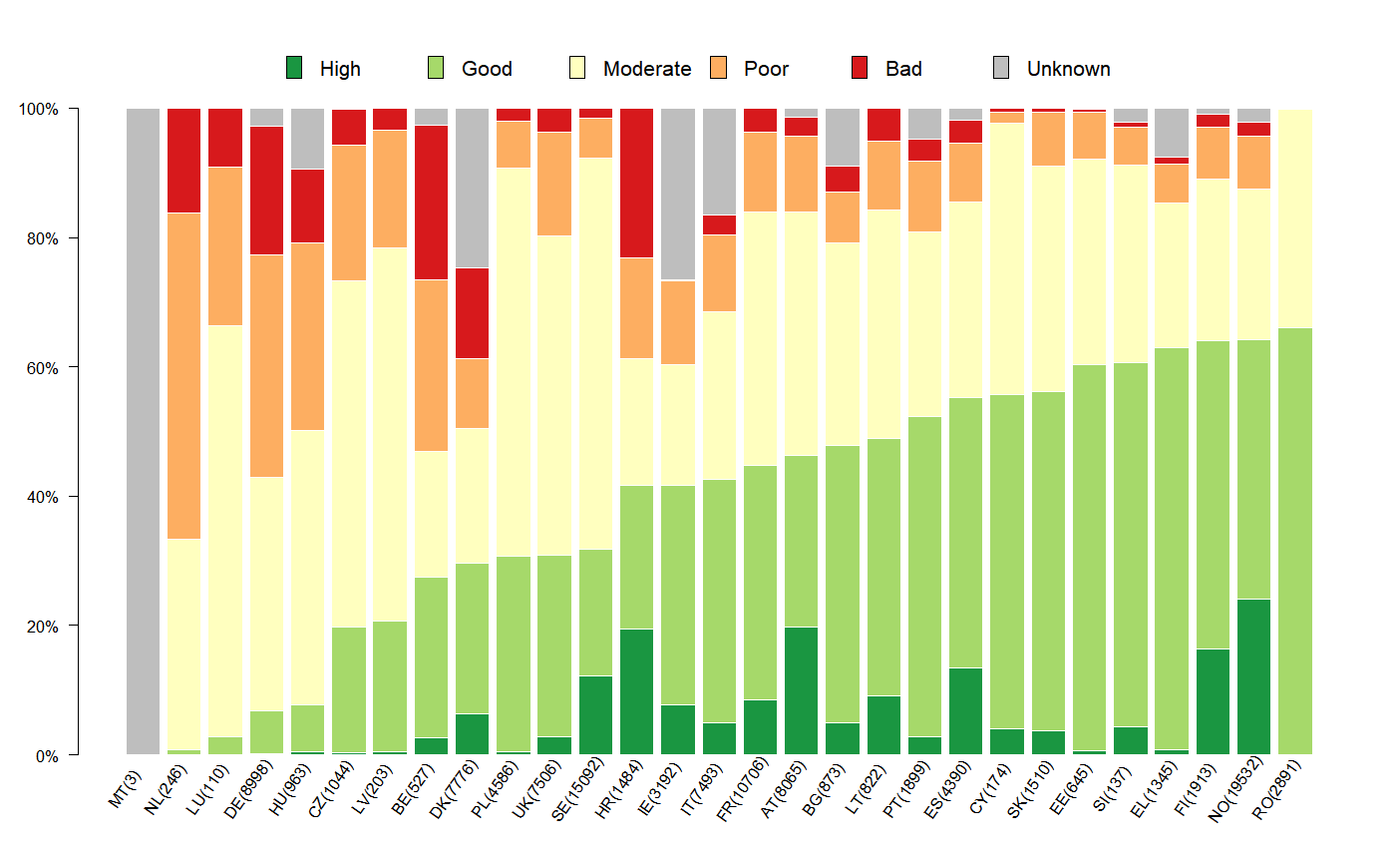

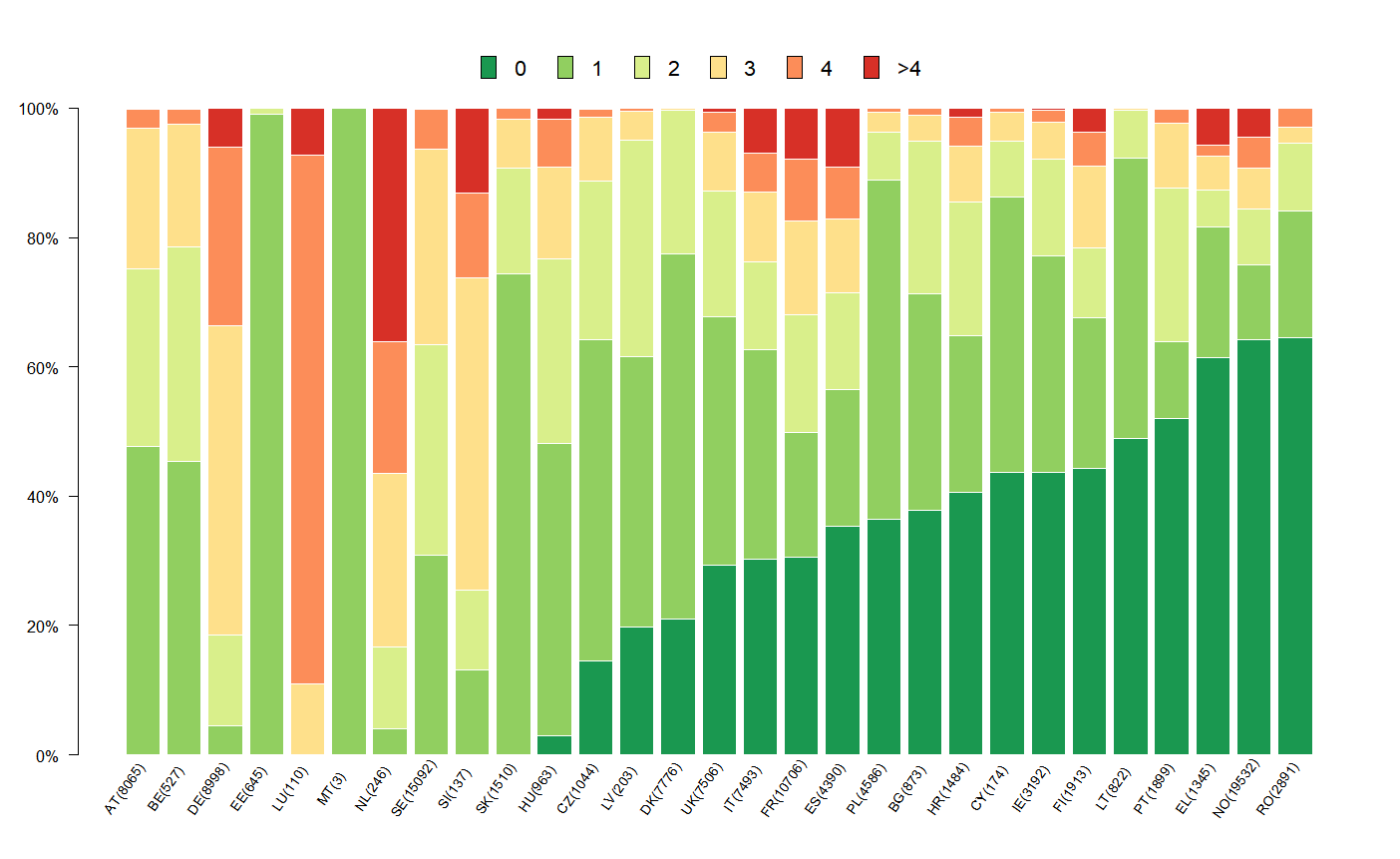


Figure SI1. Country overview of WISE-WFD information (Second RBMPs reporting round; EEA, 2020a) on river water bodies: above) ecological status classes, sorted by fraction of rivers reaching at least good ecological status; b, below) number of impact types reported for each river water body, sorted by the fraction of rivers reporting no impact.


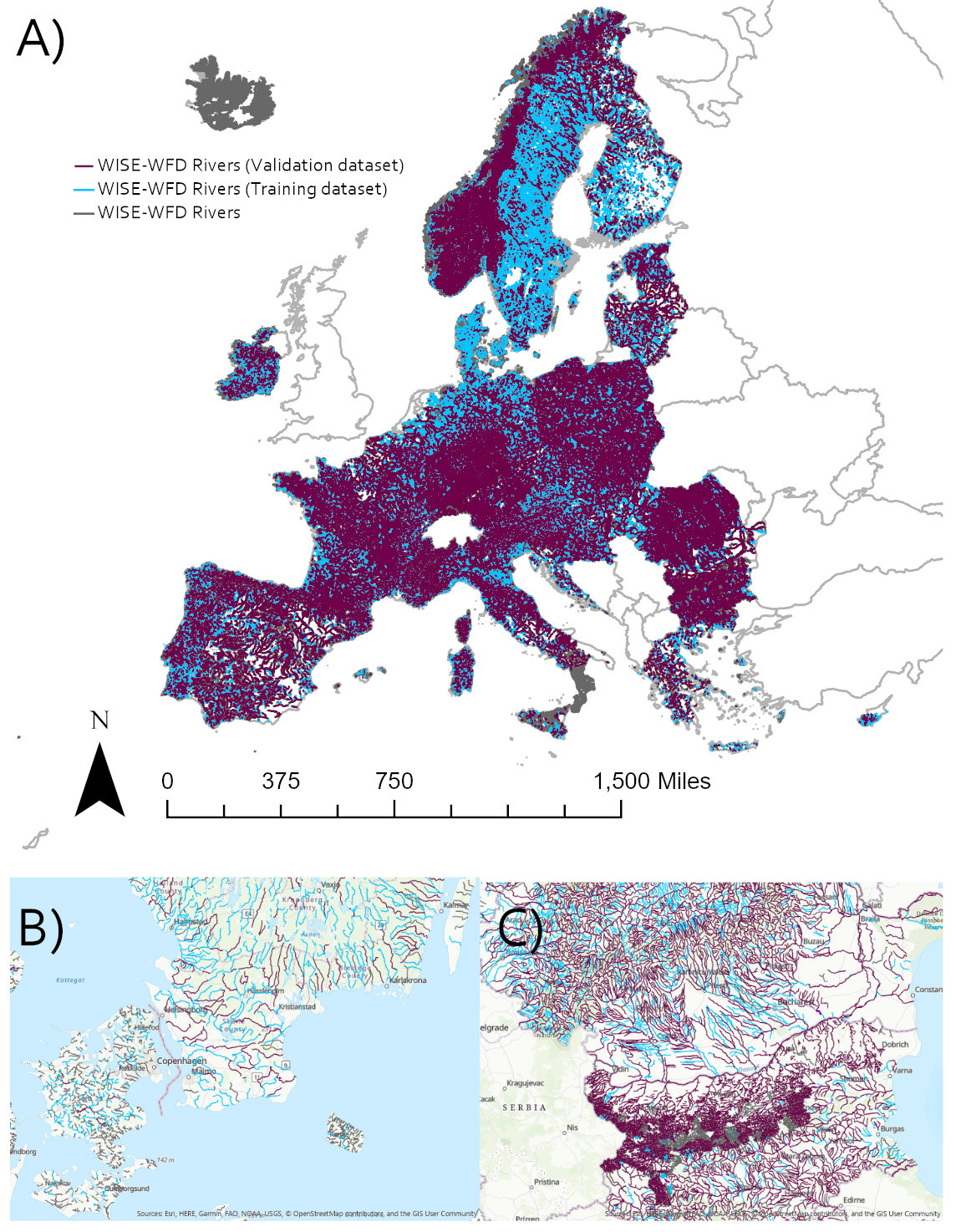


Figure SI2. Overview of WISE-WFD river water bodies used for the analysis. A) the WISE rivers geodataset (grey), overlaid with the set used for sampling rivers for model training (“WB>50” group; blue) or not (“WB<50”, used however in validation; purple).

Note to Figure SI2. Some WISE river water bodies could not be used (grey) either because of missing information (e.g. Calabria region in South Italy), or because they were not defined in CCM2 hydrological model (e.g. Iceland). In the lower panel, two blown-up detailed areas highlight how national delineation of water bodies influenced the attribution to training or validation datasets. In Denmark (Figure S2B) several small rivers were falling on the same CCM2 catchment and only one per catchment was retained; in Sweden rivers were generally longer, and most of them were used for training or validation; some coastal water bodies were not used because the paired CCM2 catchment had no reach defined in it. In Figure S2C, the lower Danube along the border between Bulgaria and Romania is shown. In Bulgaria entire ramified river systems are defined as single water bodies; for the most part they were used in the validation only (“wb<50” group) because they are large compared to CCM2 catchments. In Romania the size of rivers is closer to that of CCM2, thus they were divided more homogeneously between the two groups.

Comparison of major properties of river water bodies and in groups


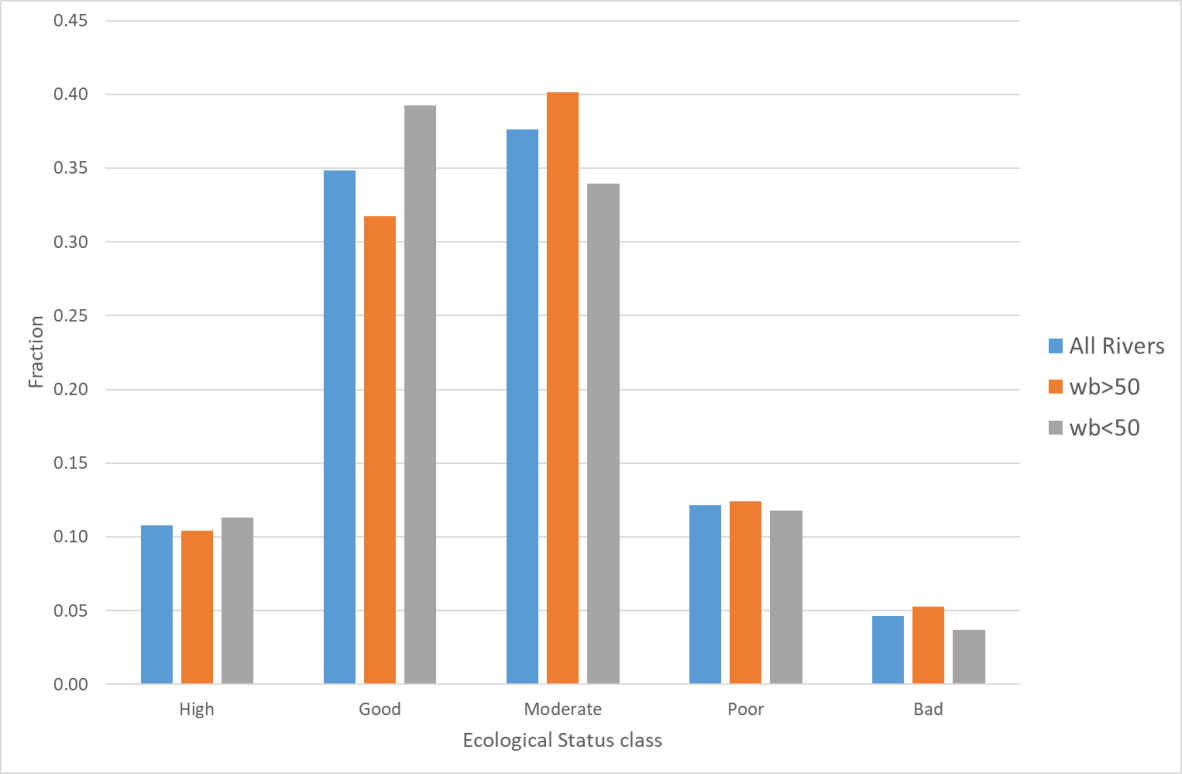


Figure SI3a. Ecological Status classes. The presence of at least good ecological status is slightly better in the wb<50 group than in the wb>50 one.


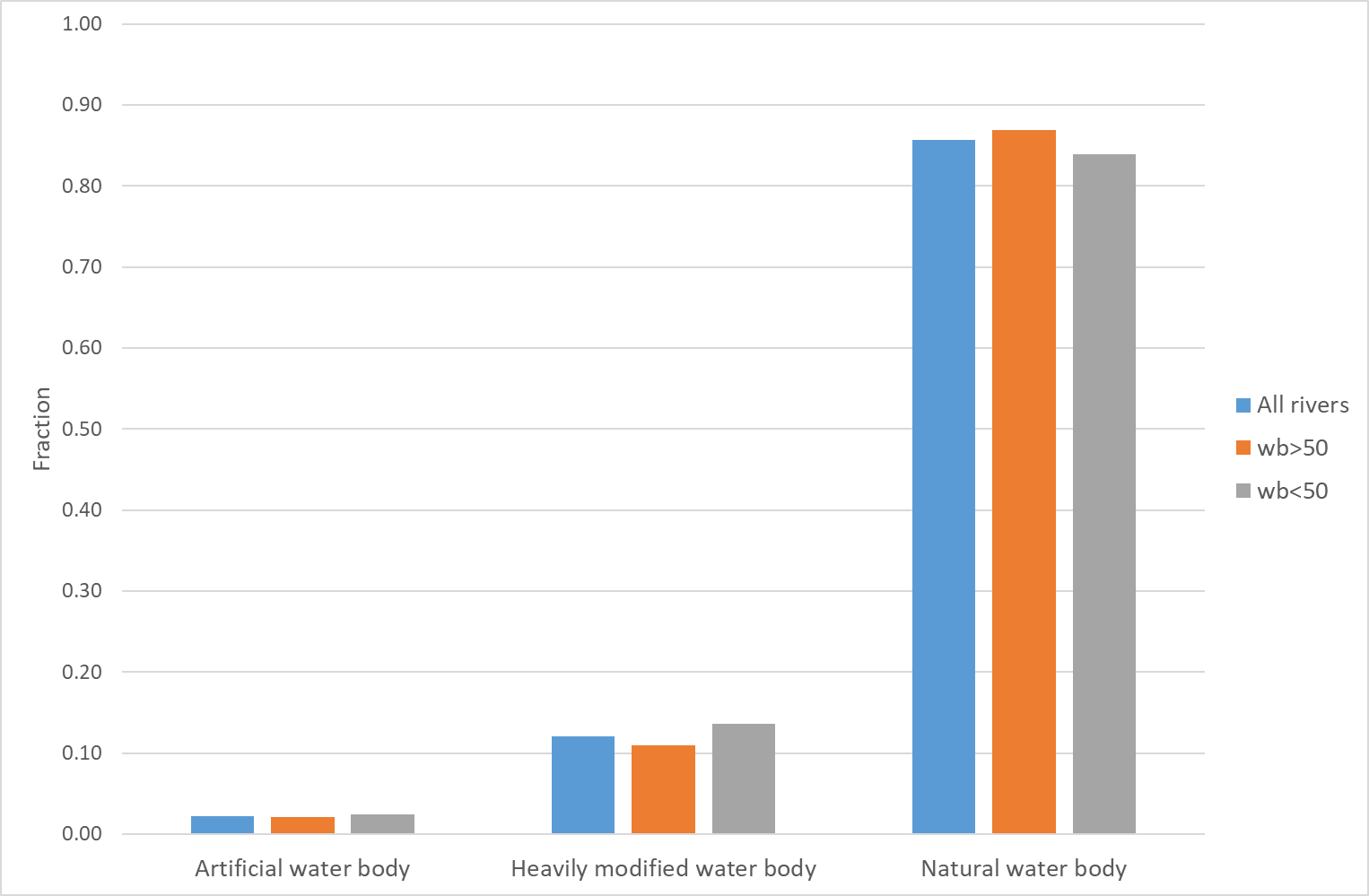


Figure SI3b. River water body types: no large changes among the groups.


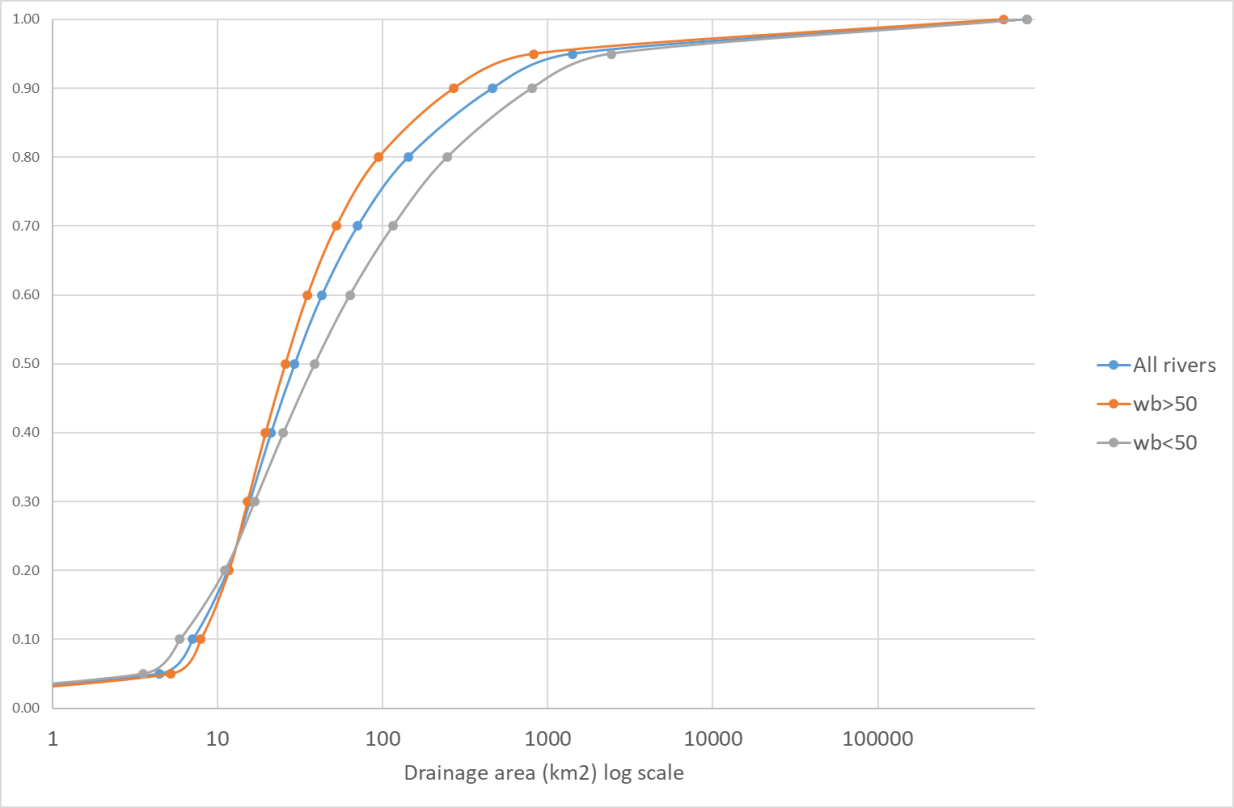


Figure SI3c. Cumulative distribution of drainage area (km2, log10 scale): slight prevalence of larger basins in the validation dataset, but large basins are present in the training dataset too.


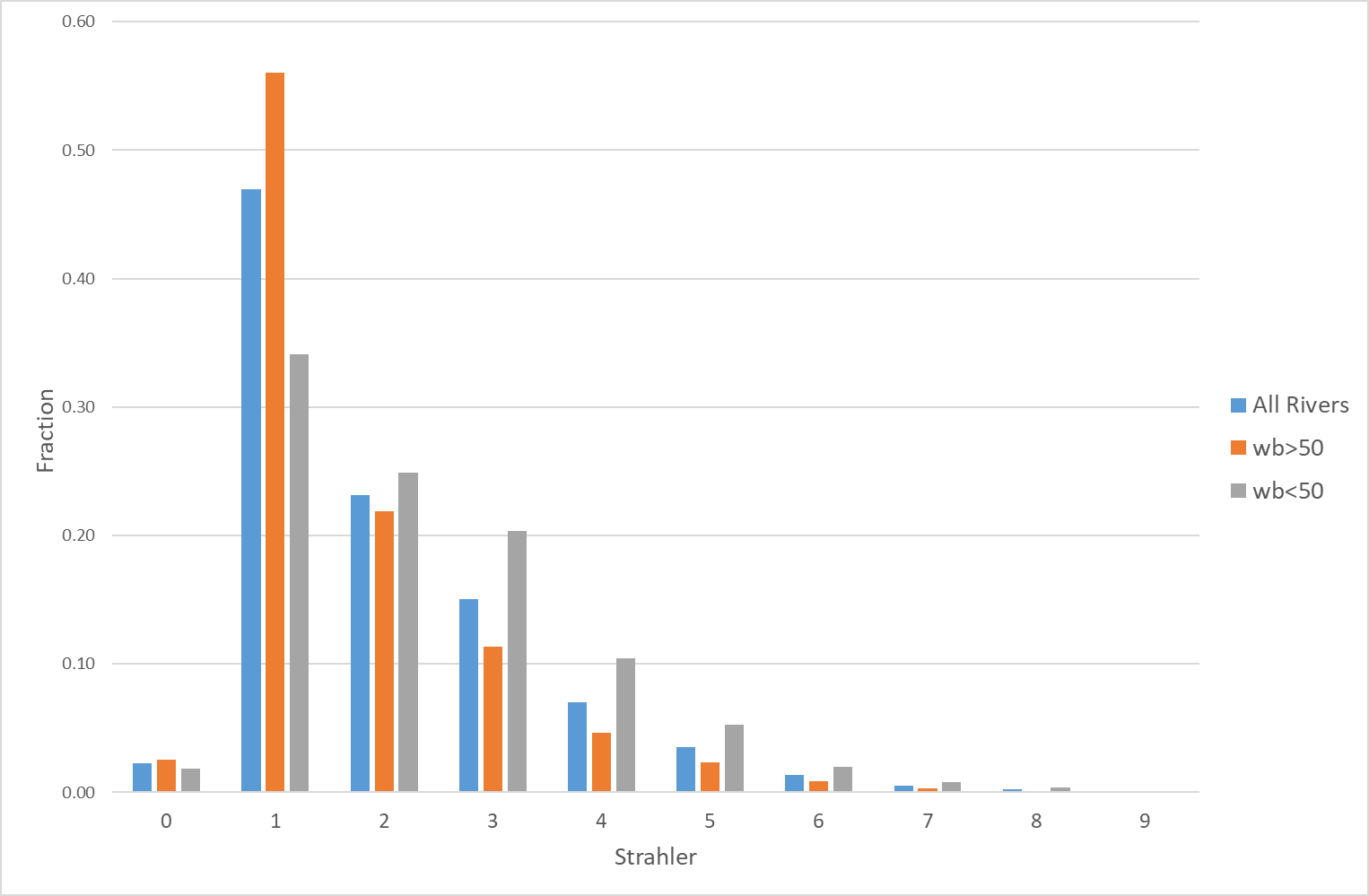


Figure SI3d. Strahler order: higher Strahler order rivers are slightly more represented in the validation dataset


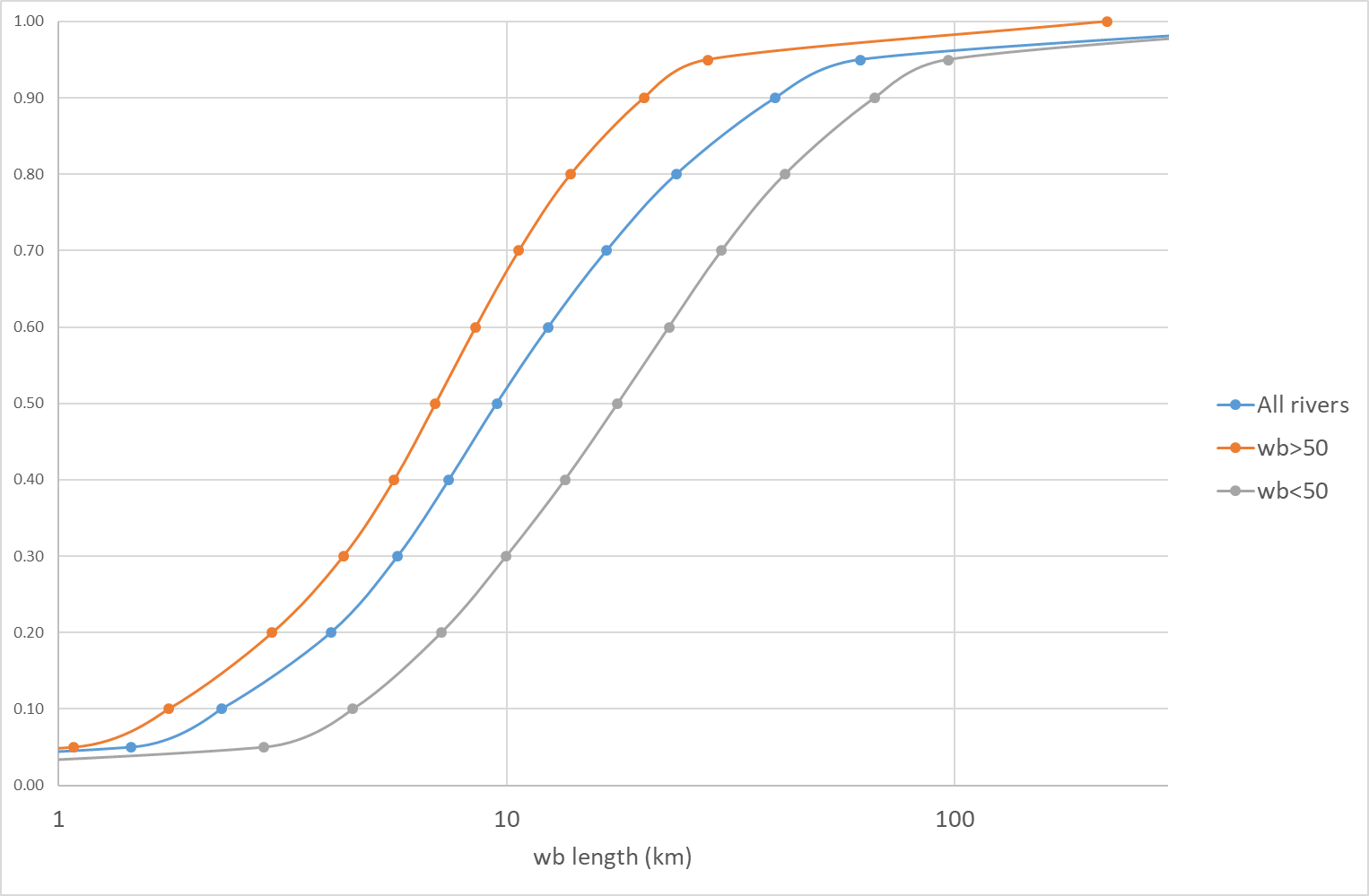


Figure SI3e. Cumulative distribution of river length (km, log10 scale): shorter river bodies prevail in the training dataset, longer ones on the validation dataset

2) Maps and density distribution of explanatory variables


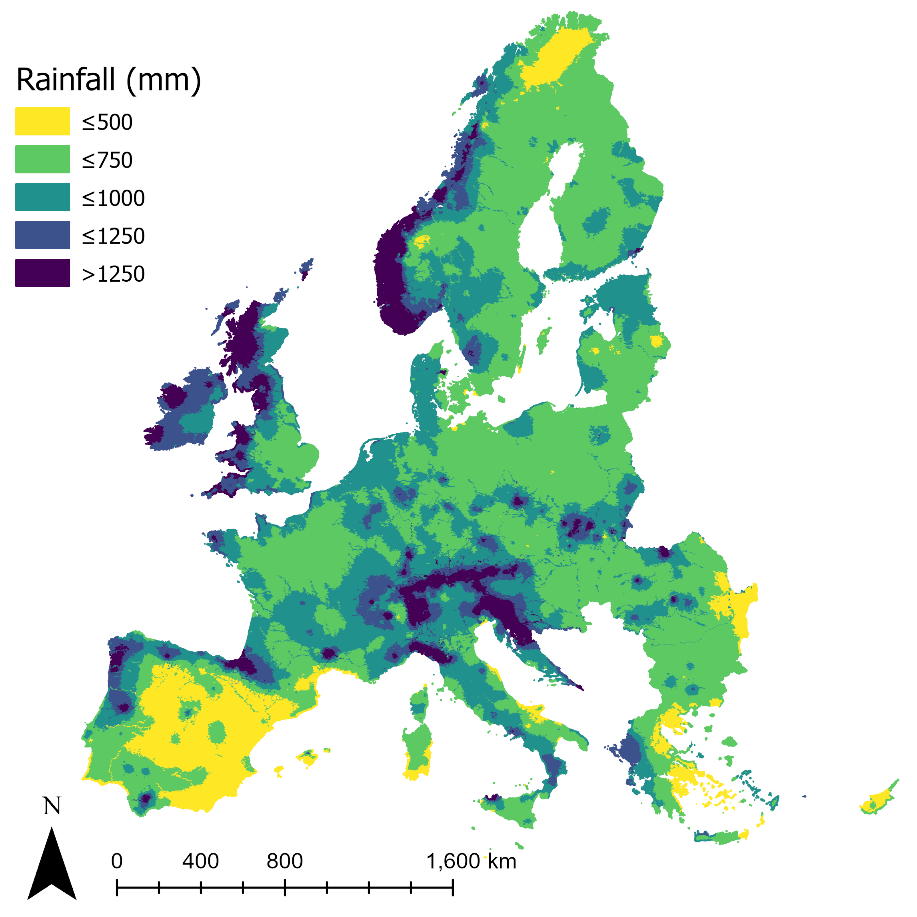

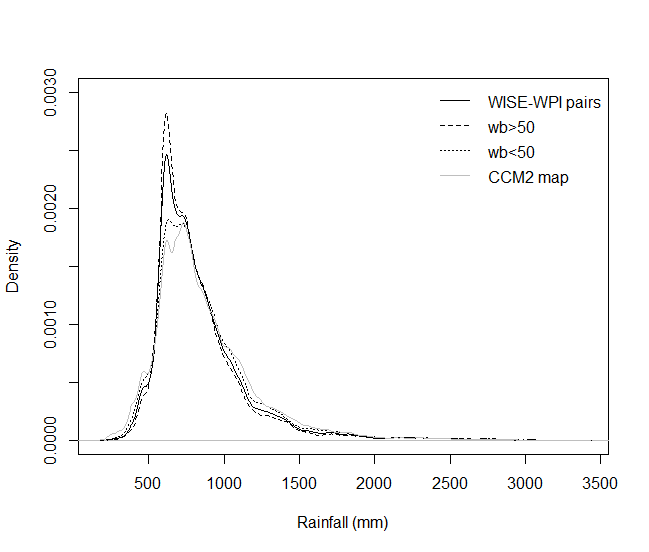


Figure SI4a. Rainfall (mm). Top: distribution in the region (CCM2 resolution). Bottom: density distribution of the WPI in WISE-WPI pairs used for model training and validation, wb>50 and wv<50 groups, as well as in the map.


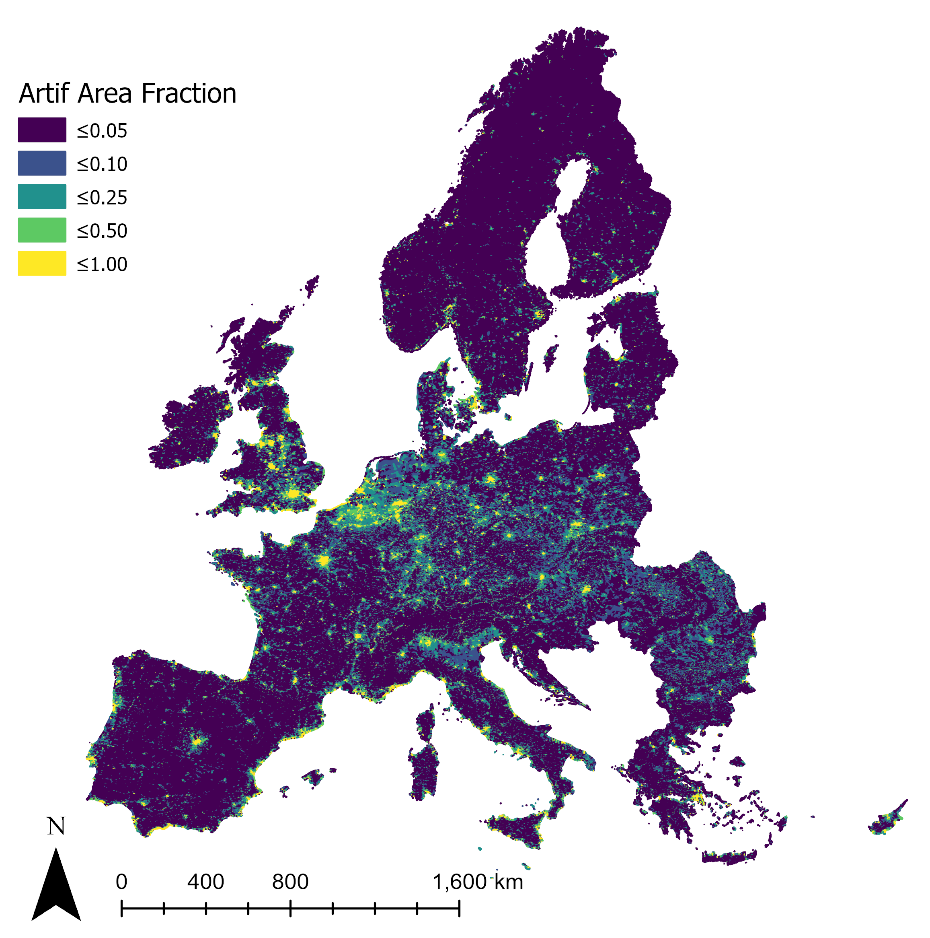

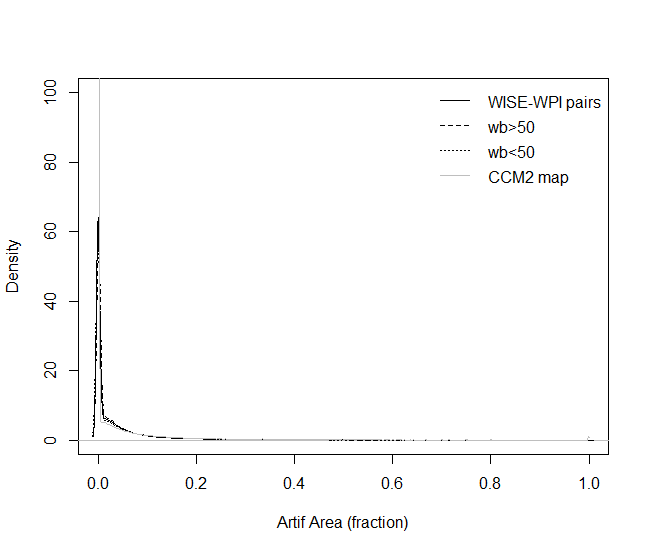


Figure SI4b. Artificial Area (fraction, 0-1). Top: distribution in the region (CCM2 resolution). Bottom: density distribution of the WPI in WISE-WPI pairs used for model training and validation, wb>50 and wv<50 groups, as well as in the map.


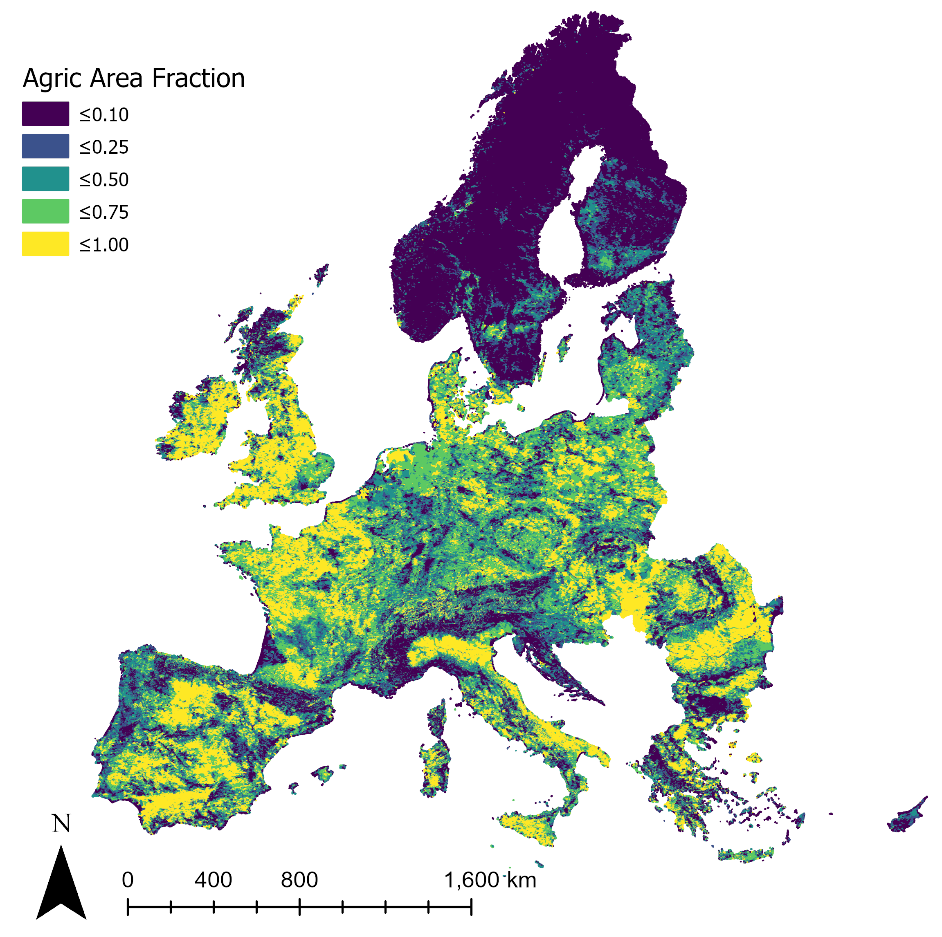

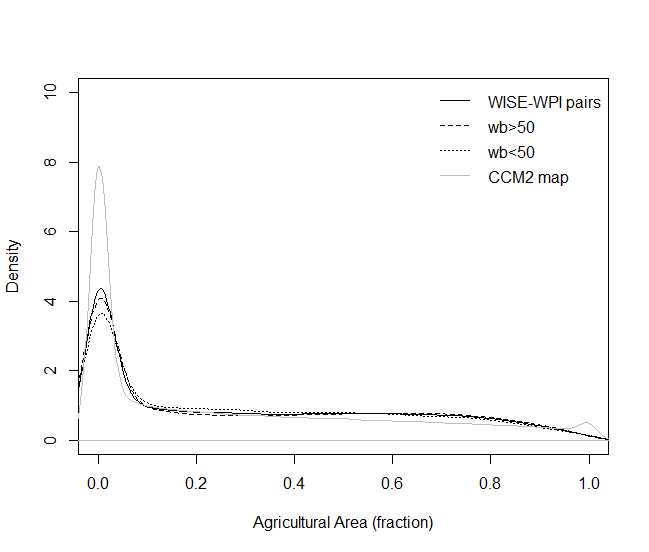


Figure SI4c. Agricultural Area (fraction, 0-1). Top: distribution in the region (CCM2 resolution). Bottom: density distribution of the WPI in WISE-WPI pairs used for model training and validation, wb>50 and wv<50 groups, as well as in the map.


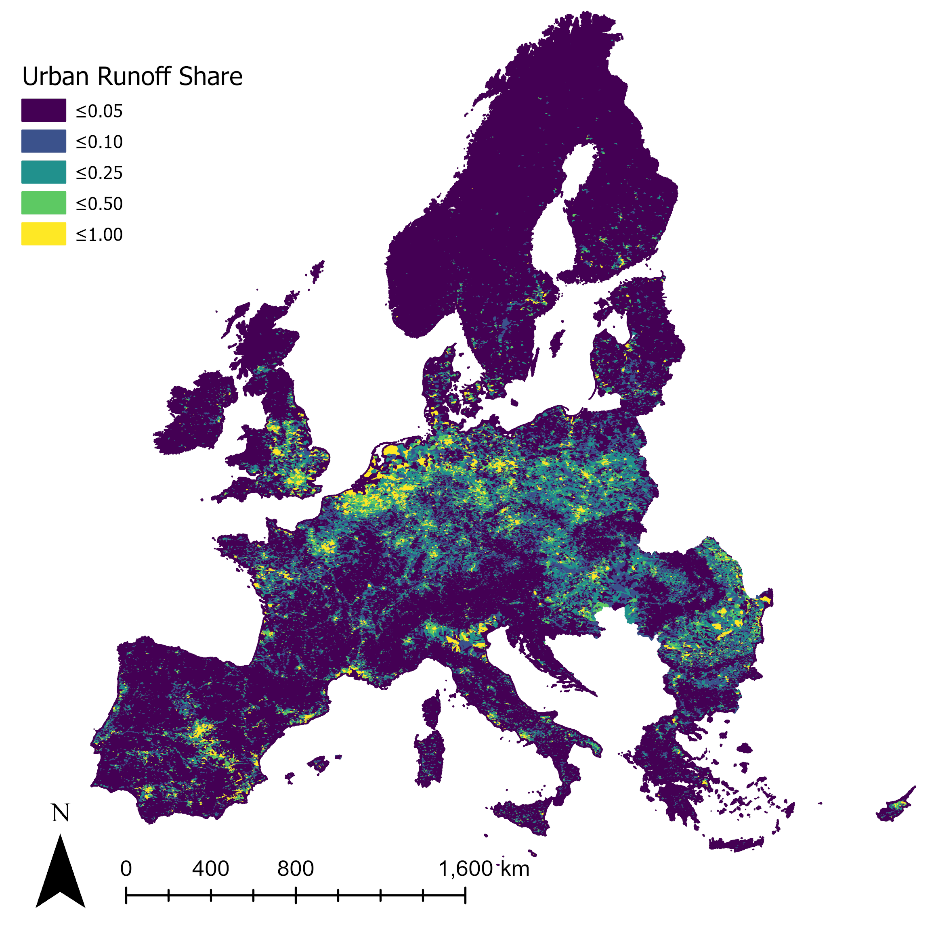

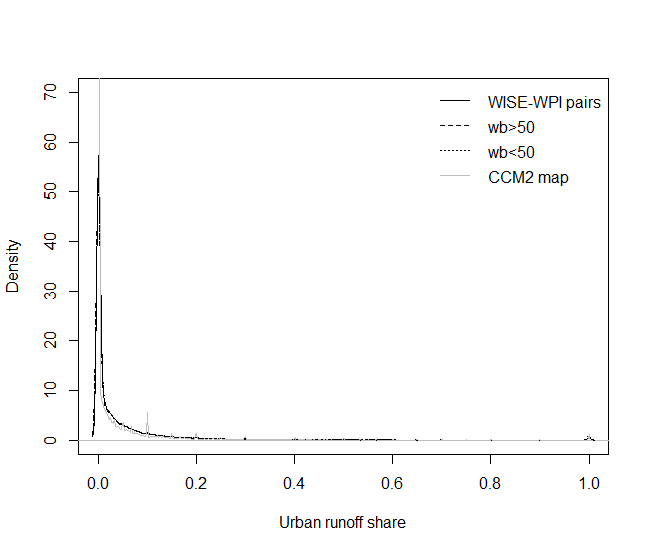


Figure SI4d. Urban runoff share (0-1). Top: distribution in the region (CCM2 resolution). Bottom: density distribution of the WPI in WISE-WPI pairs used for model training and validation, wb>50 and wv<50 groups, as well as in the map.


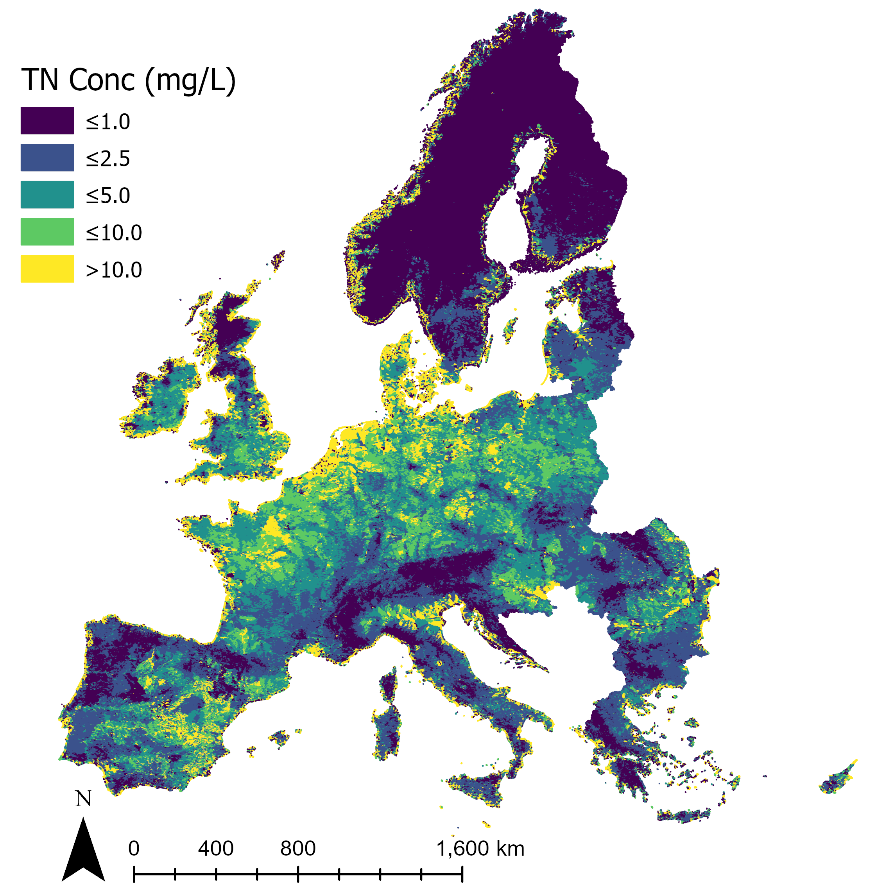

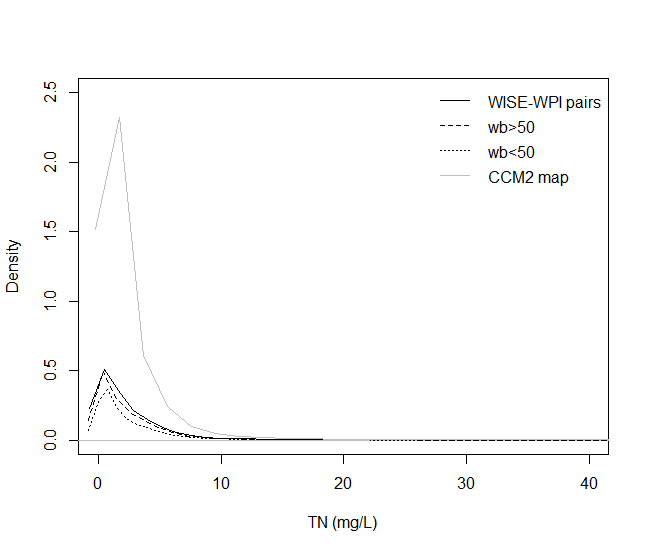


Figure SI4e. Mean Annual Total Nitrogen concentration (mg/L). Top: distribution in the region (CCM2 resolution). Bottom: density distribution of the WPI in WISE-WPI pairs used for model training and validation, wb>50 and wv<50 groups, as well as in the map. Note that in the map concentrations are limited by a minimum MAF of 0.01 m3/s and a maximum value of 1000 mg/L, which however has minimal impact on predictions.


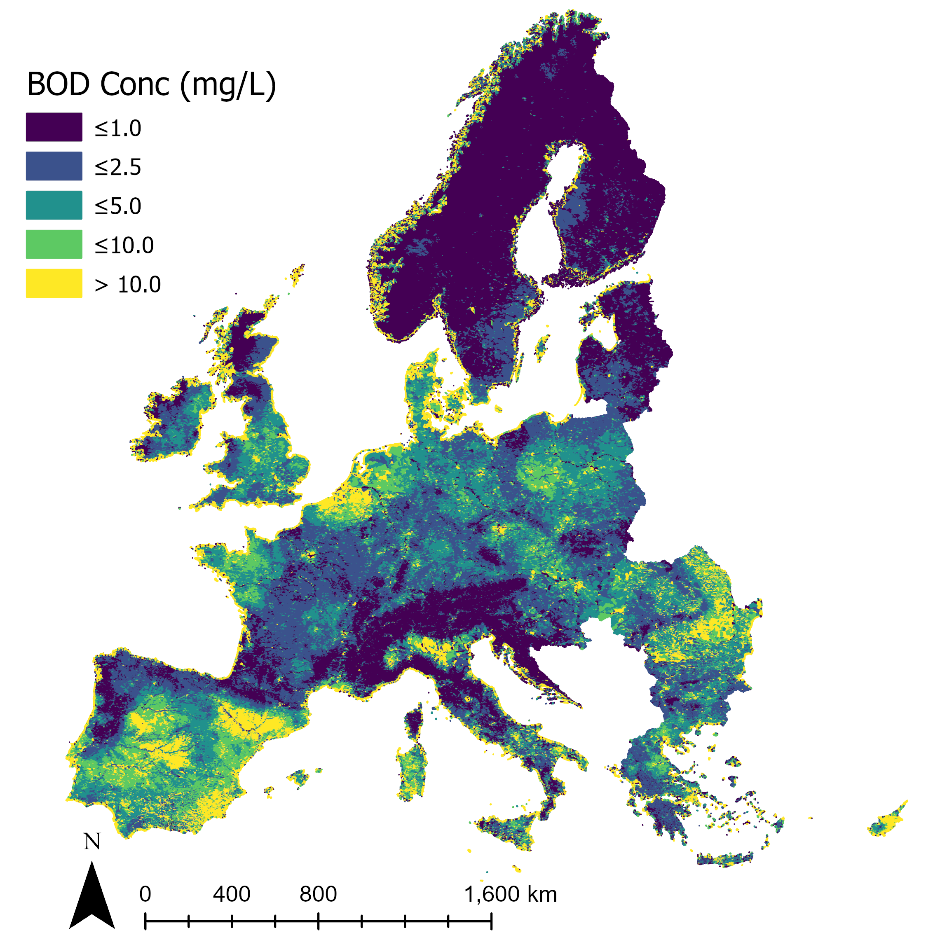

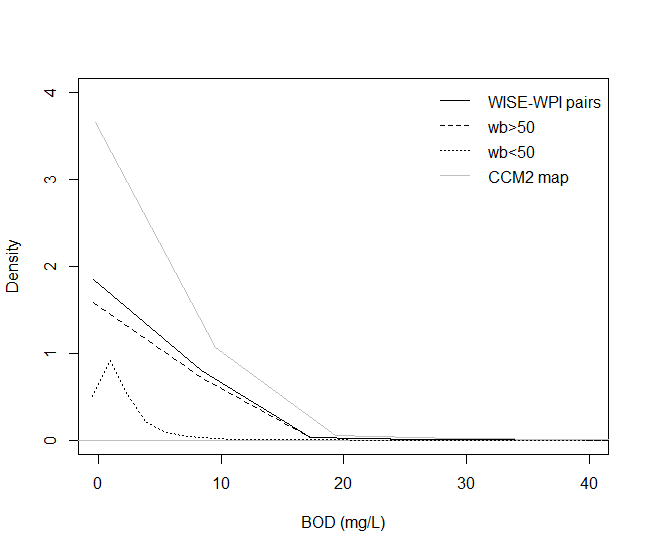


Figure SI4f. Mean Annual Biochemical Oxygen Demand concentration (BOD; mg/L). Top: distribution in the region (CCM2 resolution). Bottom: density distribution of the WPI in WISE-WPI pairs used for model training and validation, wb>50 and wv<50 groups, as well as in the map. Note that in the map concentrations are limited by a minimum MAF of 0.01 m3/s and a maximum value of 5000 mg/L, which however has minimal impact on predictions.


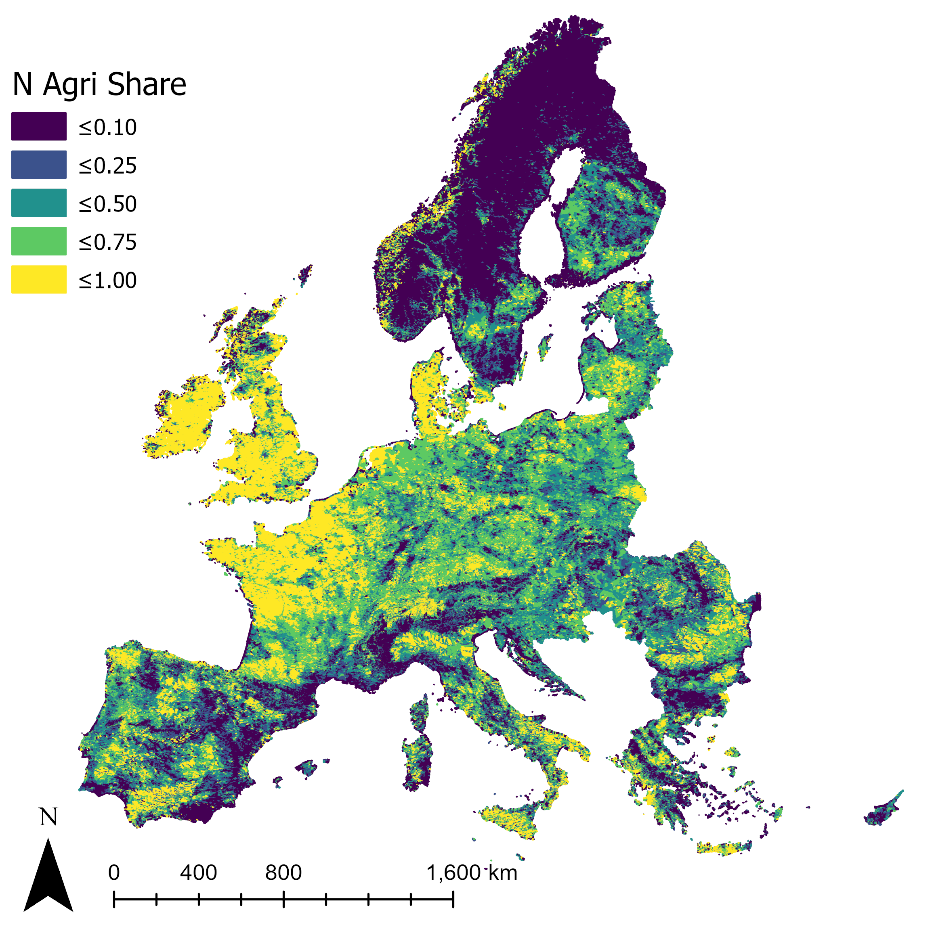


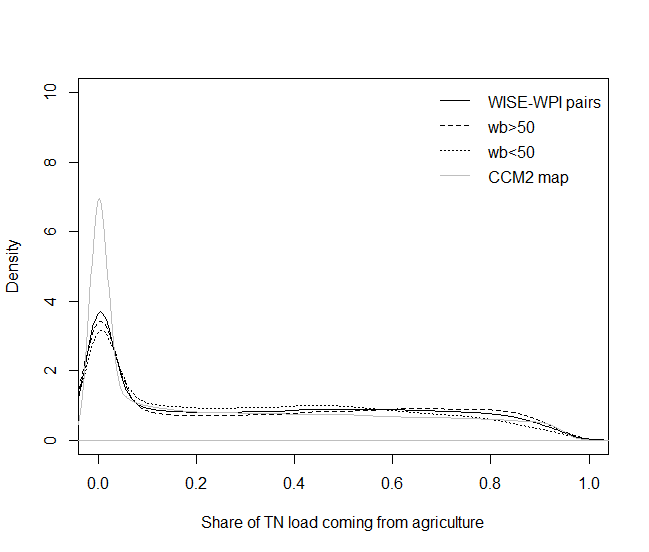


Figure SI4g. Share of total nitrogen load coming from agriculture (0-1). Top: distribution in the region (CCM2 resolution). Bottom: density distribution of the WPI in WISE-WPI pairs used for model training and validation, wb>50 and wv<50 groups, as well as in the map.


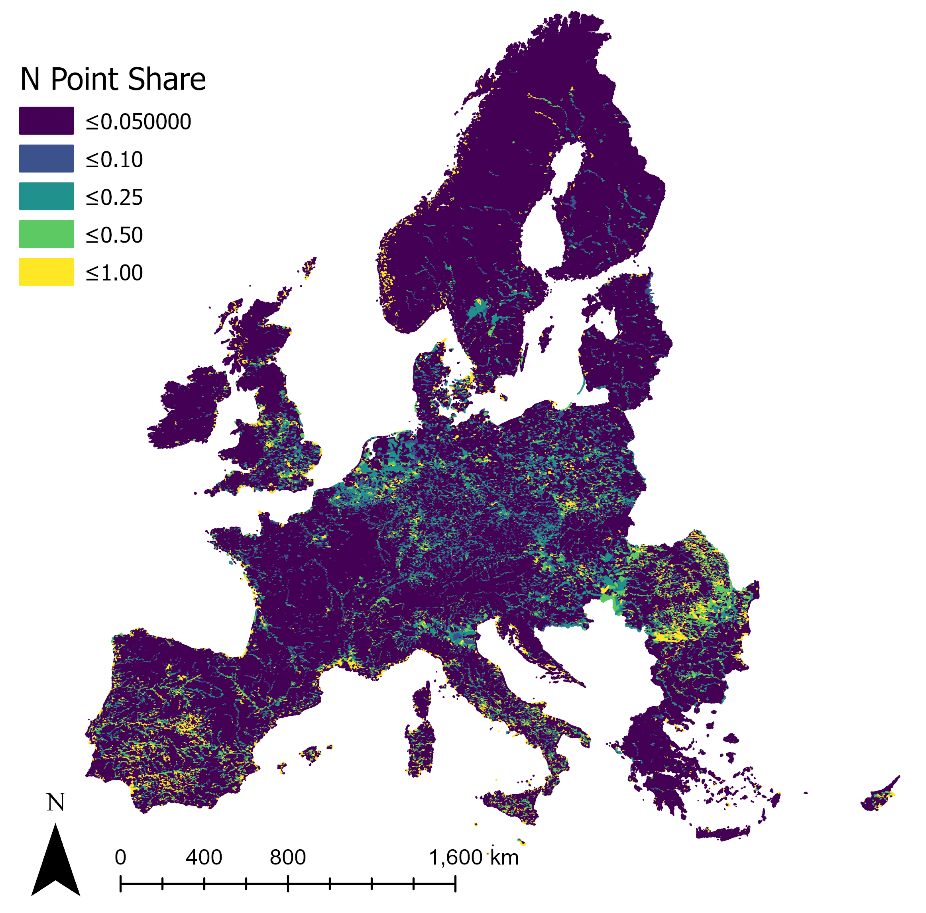


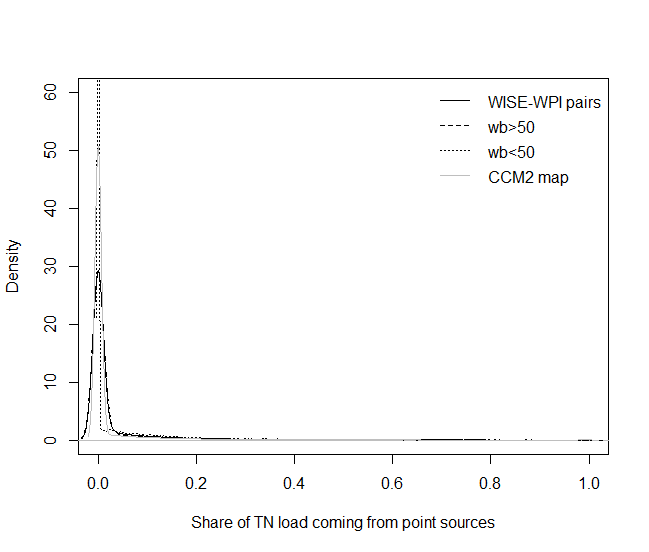


Figure SI4h. Share of total nitrogen load coming from point sources (0-1). Top: distribution in the region (CCM2 resolution). Bottom: density distribution of the WPI in WISE-WPI pairs used for model training and validation, wb>50 and wv<50 groups, as well as in the map. Note that this WPI may be underrepresented in the wb>50 group, although all the range is represented.


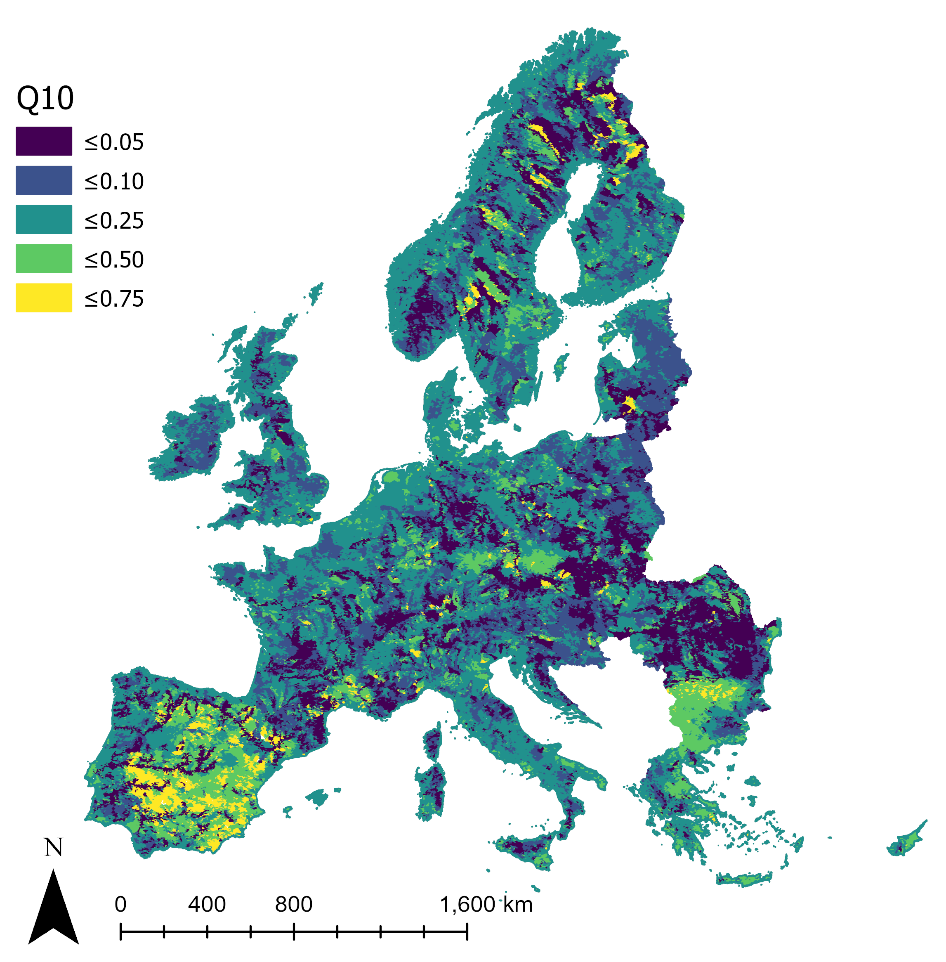


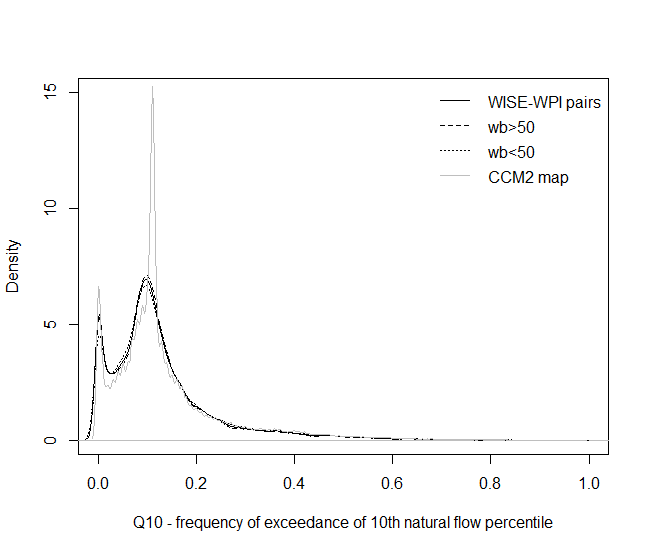


Figure SI4i. Low flow exceedance (Q10, frequency of exceedance of 10^th^ percentile of natural flow, 0-1). Top: distribution in the region (CCM2 resolution). Bottom: density distribution of the WPI in WISE-WPI pairs used for model training and validation, wb>50 and wv<50 groups, as well as in the map. Note that this WPI was set at 0.11 in coastal area for which the original WPI could not be assessed.


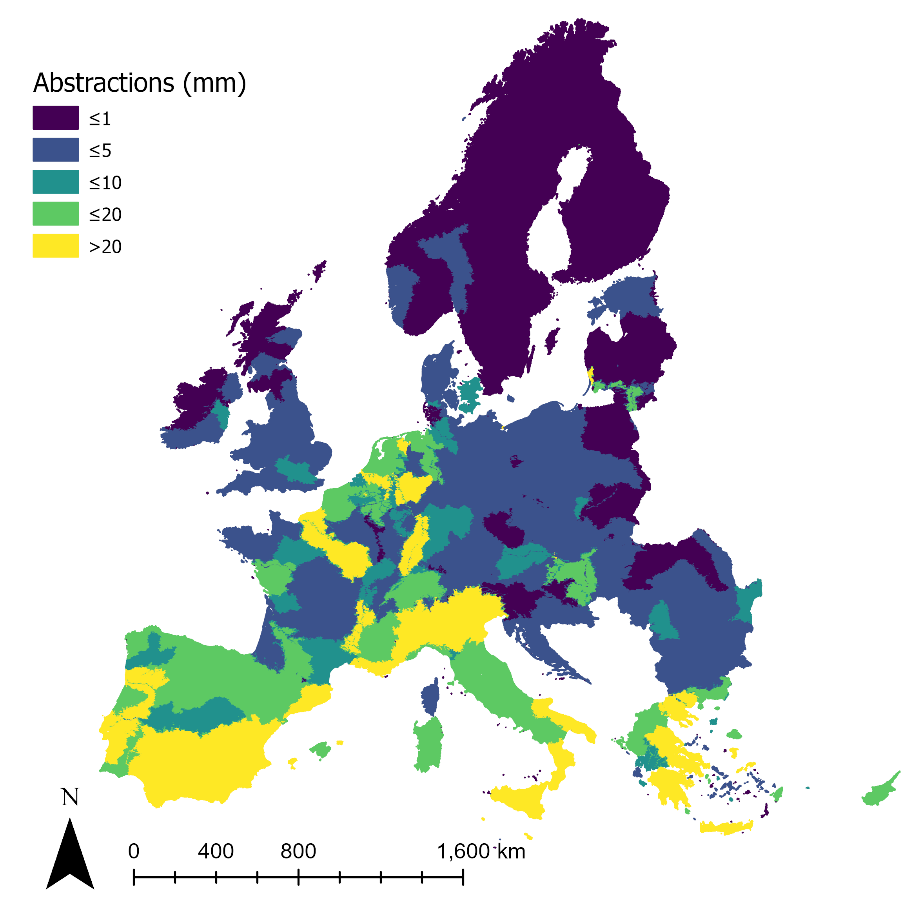


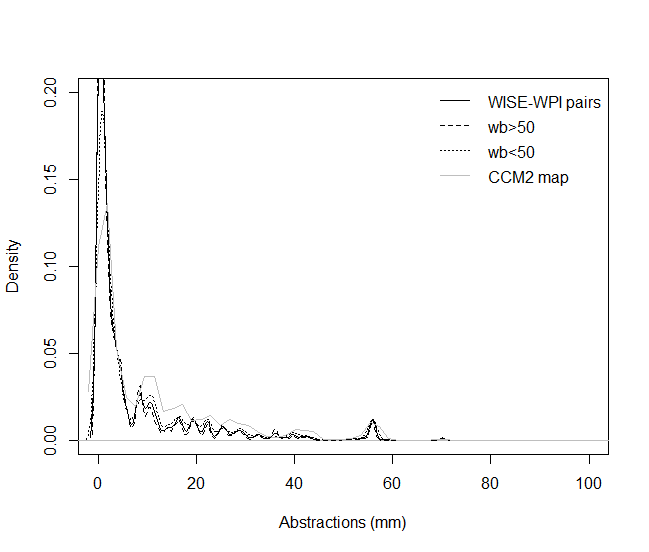


Figure SI4j. Net abstractions (mm). Top: distribution in the region (CCM2 resolution). Bottom: density distribution of the WPI in WISE-WPI pairs used for model training and validation, wb>50 and wv<50 groups, as well as in the map. Note that abstractions were calculated per river basin.


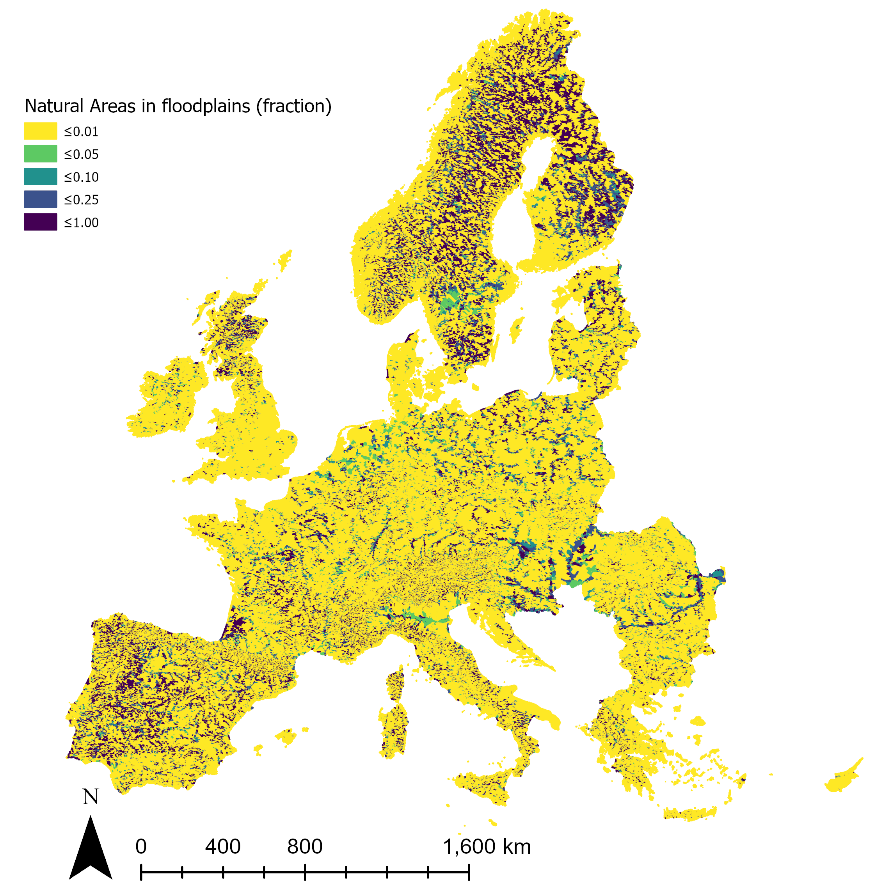


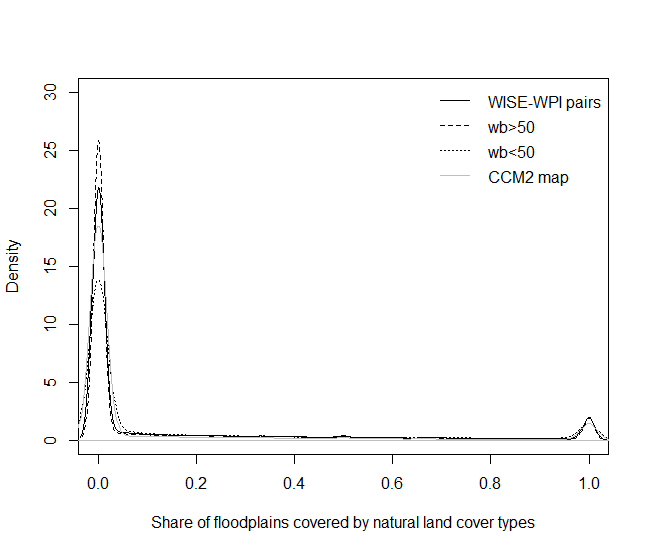


Figure SI4k. Share of riparian and floodplain areas covered with natural land use types (fraction, 0-1). Top: distribution in the region (CCM2 resolution). Bottom: density distribution of the WPI in WISE-WPI pairs used for model training and validation, wb>50 and wv<50 groups, as well as in the map.


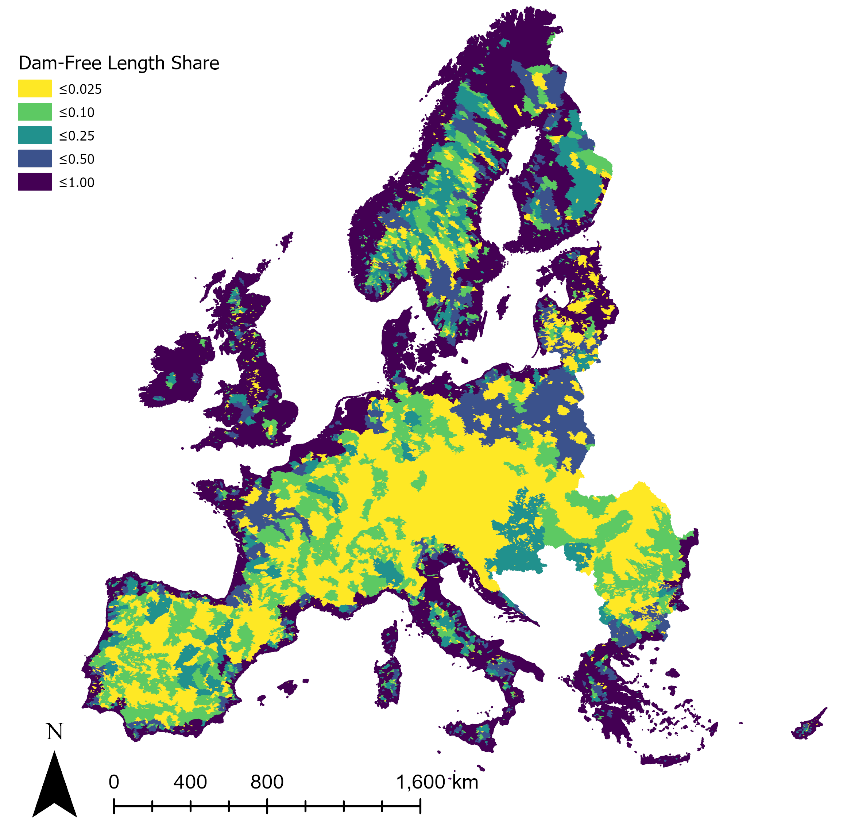


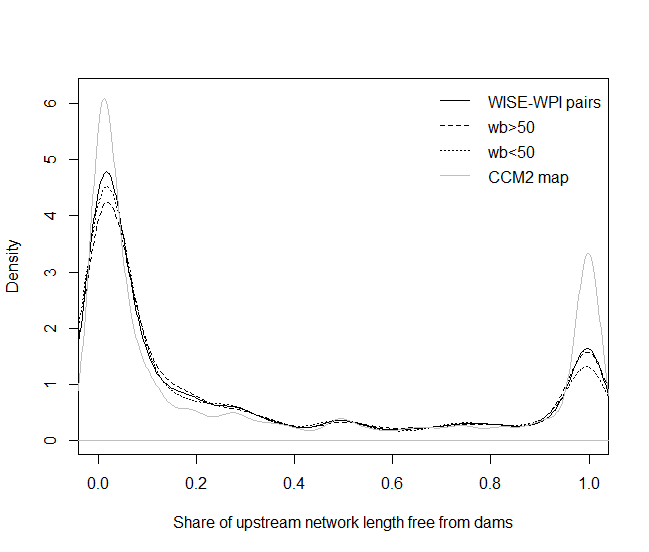


Figure SI4l. Share of upstream river network free from dams (fraction, 0-1). Top: distribution in the region (CCM2 resolution). Bottom: density distribution of the WPI in WISE-WPI pairs used for model training and validation, wb>50 and wv<50 groups, as well as in the map.

3) WPIS correlations in the training dataset (wb>50 group)


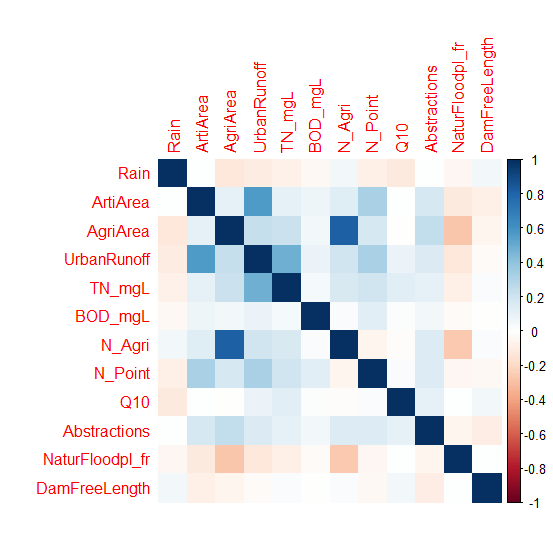


Figure SI5. Correlation plot of selected WPIs – training dataset (wb>50 group).

The highest Pearson’s correlations were:

N_Agri and AgriArea: 0.82

UrbanRunoff and ArtiArea: 0.56

ArtiArea and N_Point: 0.32

UrbanRunoff and TN: 0.49

AgriArea and NaturFloodpl_fr: -0.28

N_Agri and NaturFloodpl_fr: -0.27

Table SI1. Ranges and rank of coefficients of the logistic regressions for predicting the probability of failing to achieve good Ecological Status, or occurrence of nutrient pollution; organic pollution; chemical pollution; altered hydrology; altered morphology; and no impact. The values are minimum and maximum of 100 repetitions; in brackets is indicated the rank of importance of the explanatory variable according to AIC.

|  | Failing good Ecological Status | Nutrient pollution | Organic pollution | Chemical pollution | Altered hydrology | Altered morphology | No impact |
| --- | --- | --- | --- | --- | --- | --- | --- |
| *β_0_* - intercept | -1.001;  -0.969 | -0.729; -0.508 | -1.272; -1.196 | -0.002;  0.180 | -1.179; -0.960 | -0.485; -0.435 | 0.391; 0.513 |
| *β_1_* Rain |  | -0.0009; -0.0006 (3) |  | -0.0002;  0.0000 (5) | 0.0009; 0.0012 (2) |  |  |
| *β_2_* ArtiArea | 5.387; 5.909 (2) | 1.315; 1.975 (5) | 1.678; 2.480 (4) |  | 0.673; 1.453 (5) | 2.358;  2.926 (1) | -6.326; -5.331 (2) |
| *β_3_* AgriArea | 2.194; 2.294 (1) |  |  |  |  | 0.584;  0.682 (2) | -1.279;  -1.084 (1) |
| *β_4_* TN_mgL |  |  | -0.0051;  -0.0015 (5) |  |  |  |  |
| *β_5_* NShareAgri |  | 2.113; 2.320 (1) | 1.691; 1.859 (1) |  |  |  |  |
| *β_6_* NSharePoint | 1.062; 1.372 (3) | 2.194; 2.669 (2) | 2.458; 2.914 (2) |  |  |  | -1.122;  -0.404 (5) |
| *β_7_* UrbRunoffShare |  | 0.589; 1.058 (6) |  | 1.350; 1.791 (2) |  | 0.490; 0.773 (5) |  |
| *β_8_* BOD_mgL |  |  |  |  |  | -0.025;-0.017 (4) |  |
| *β_9_* Q10 |  |  |  | 0.153; 0.658 (4) | -1.420; -0.558 (6) |  |  |
| *β_10_* Abstraction | -0.004;  -0.002 (4) |  | 0.016; 0.020 (3) | 0.013; 0.017 (3) | 0.024; 0.032 (1) | 0.009; 0.012 (3) | 0.014; 0.019 (3) |
| *β_11_* NaturalFlood_RipFr | -0.101;  -0.006 (5) |  |  |  | 0.646; 1.045 (3) |  |  |
| *β_12_* DamFreeLength |  | -0.374;  -0.265 (4) |  | -1.020;  -0.864 (1) | -0.560;  -0.312 (4) |  | 0.205; 0.371 (4) |

5) ROC curves of logistic regressions

**Failing good ecological status**

| 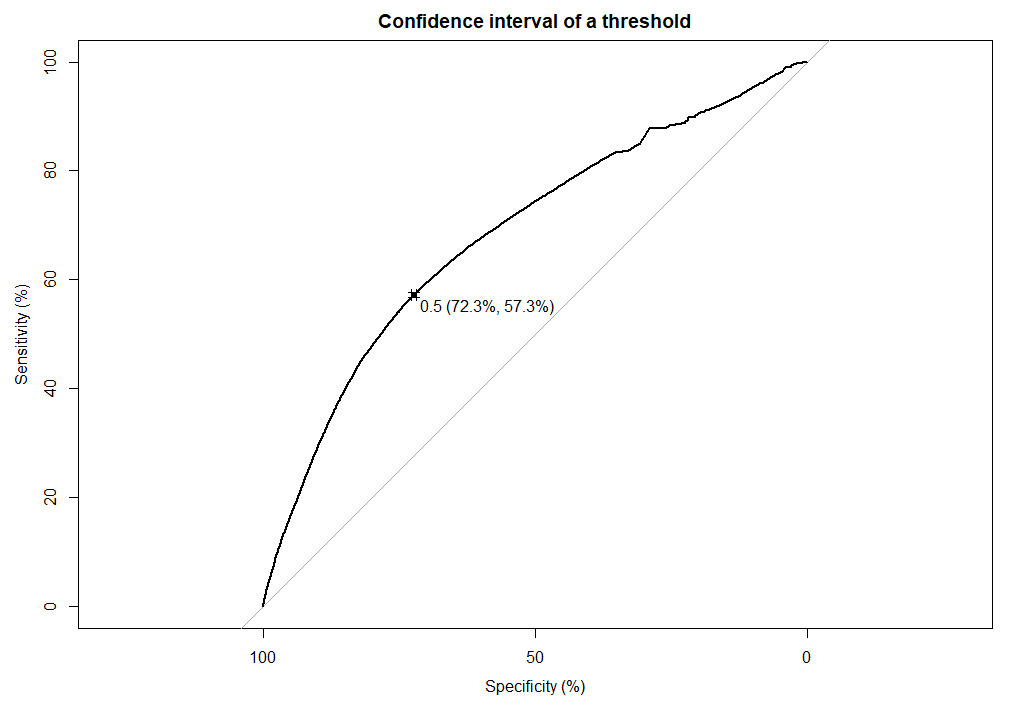 | 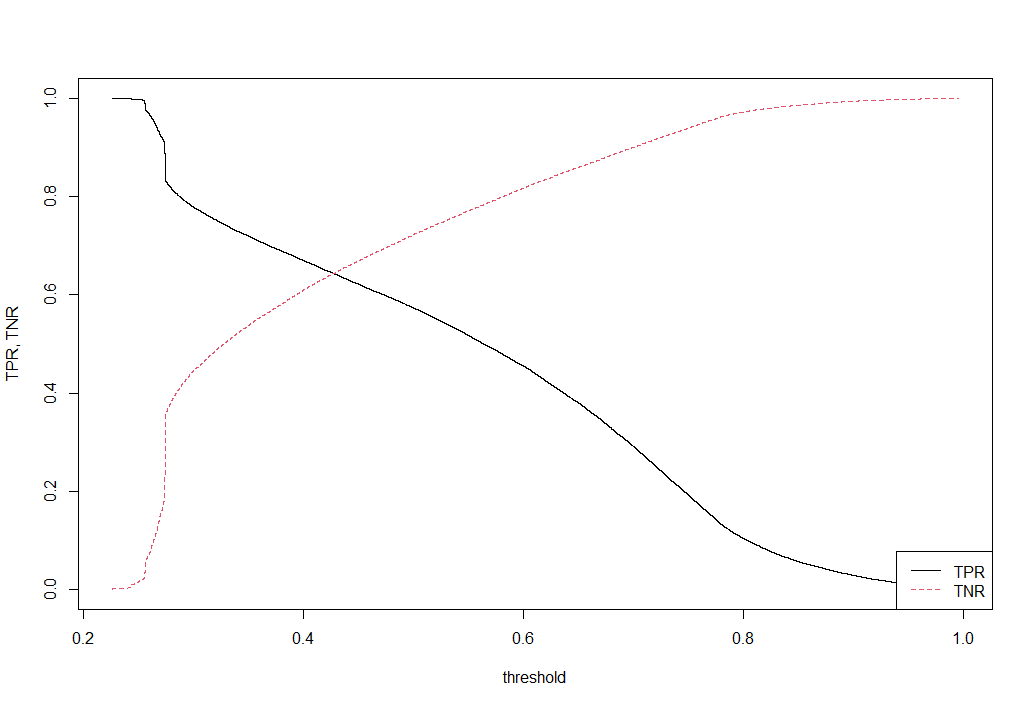 |
| --- | --- |

# Nutrient pollution

| 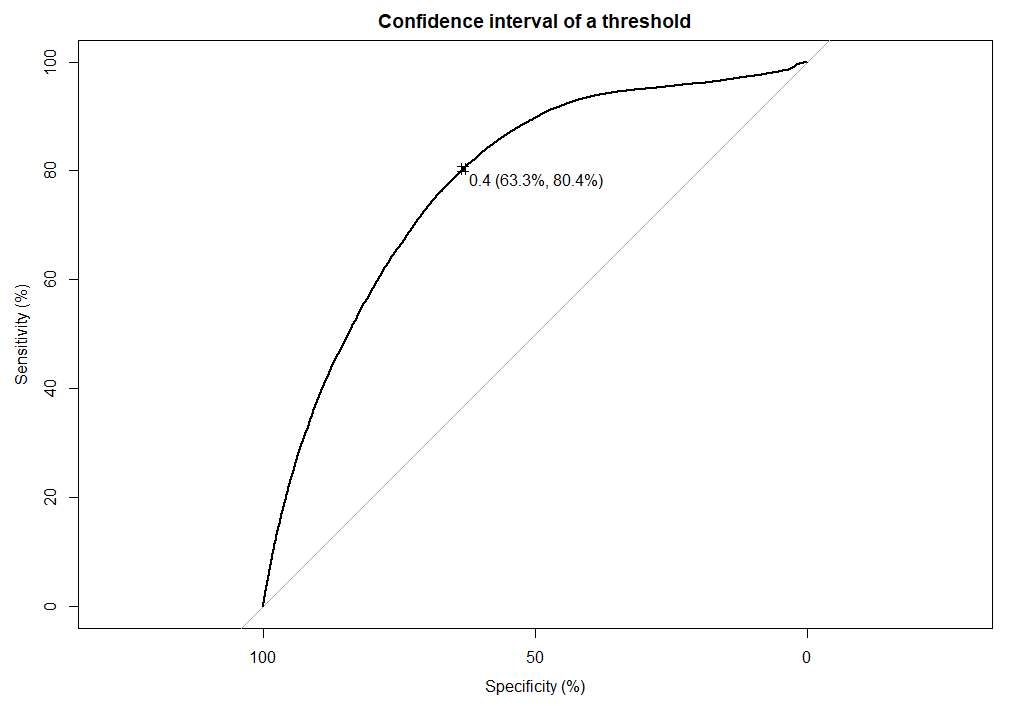 | 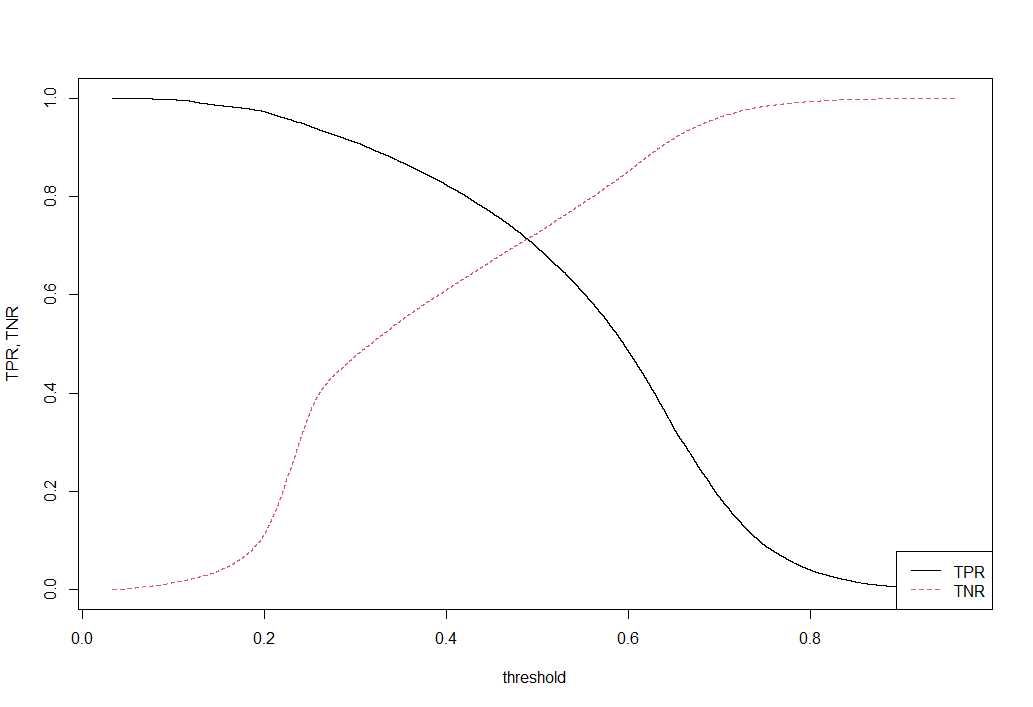 |
| --- | --- |

# Organic pollution

| 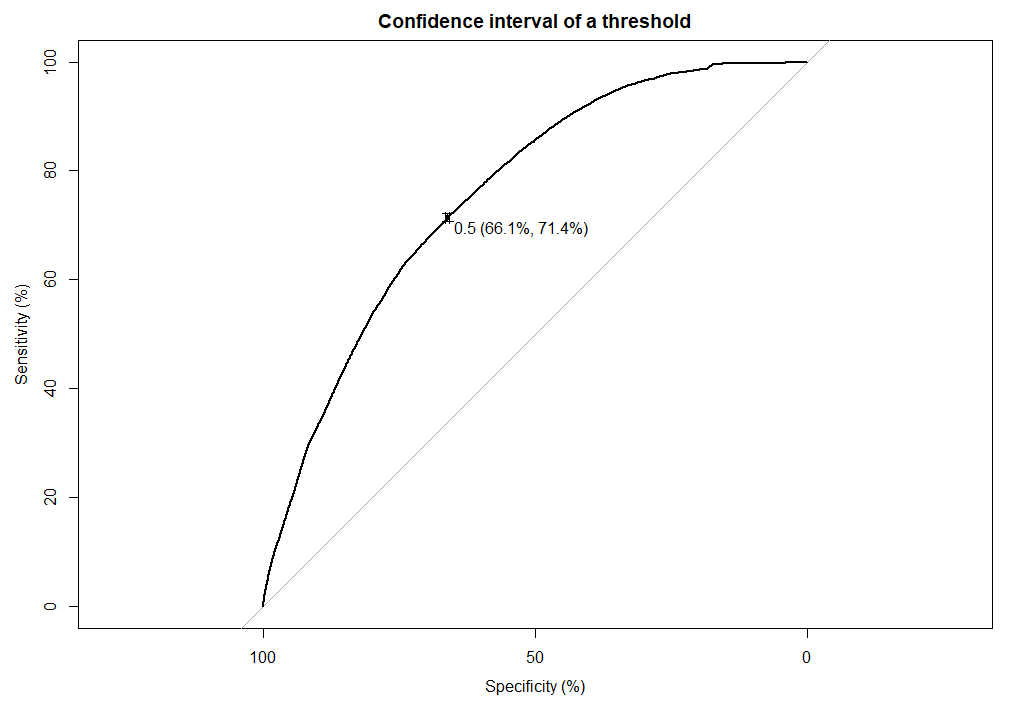 | 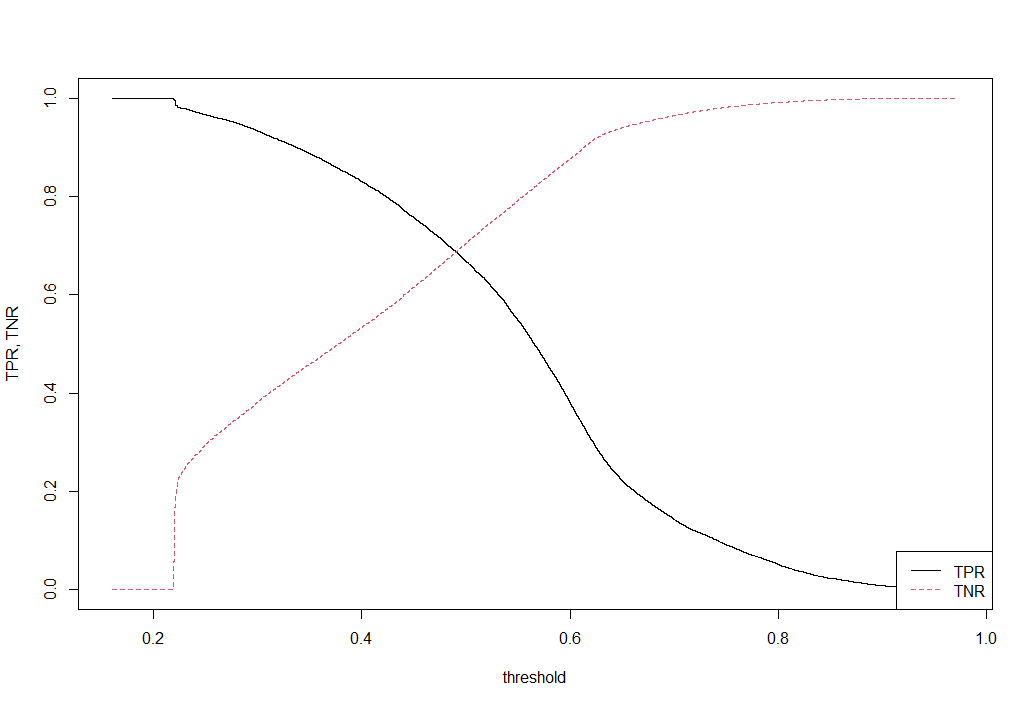 |
| --- | --- |

**Chemical pollution**

| 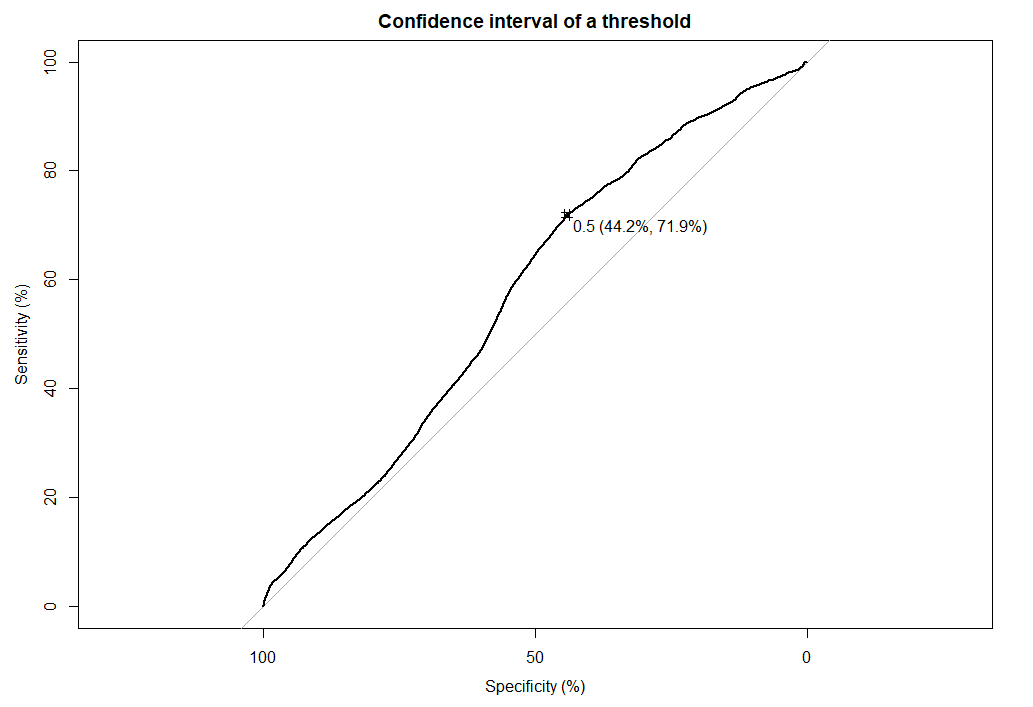 | 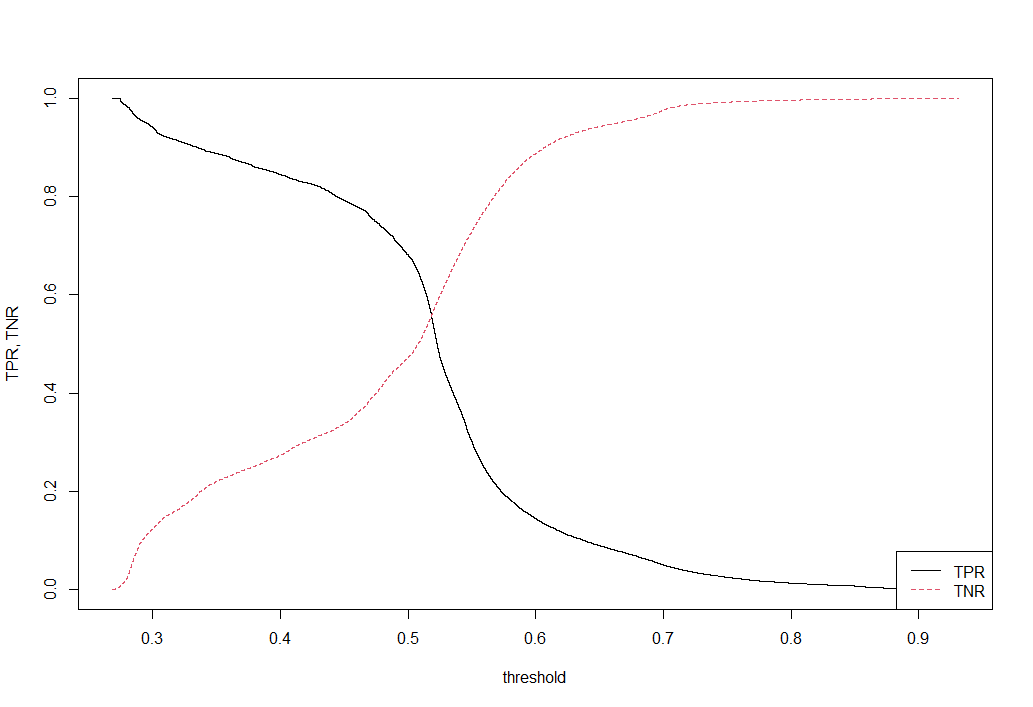 |
| --- | --- |

# Altered hydrology

| 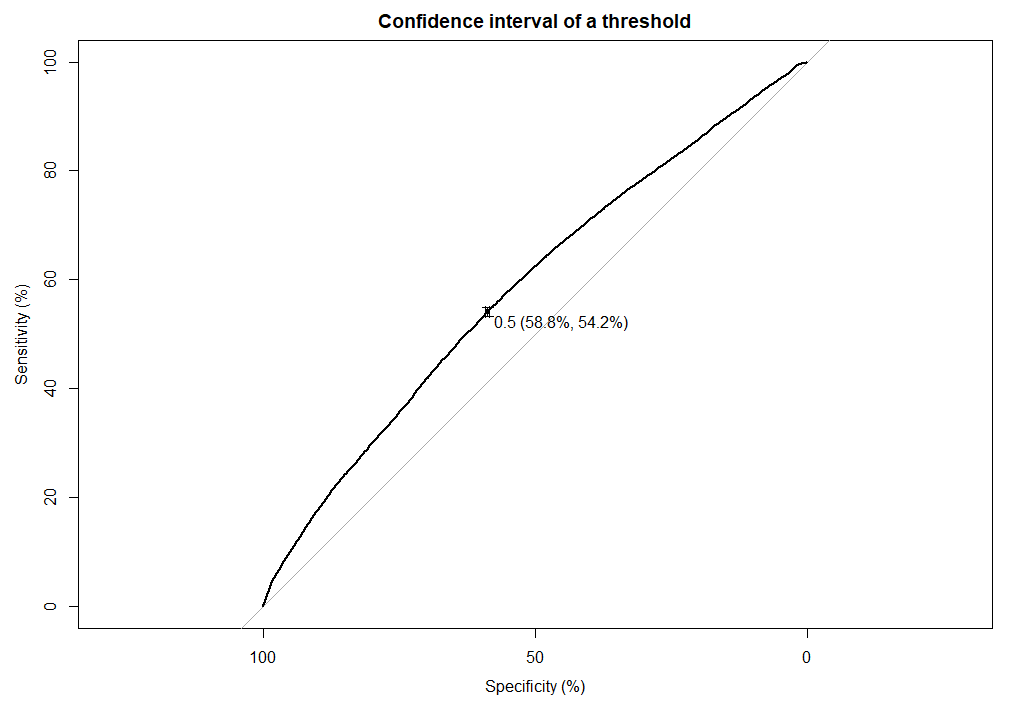 | 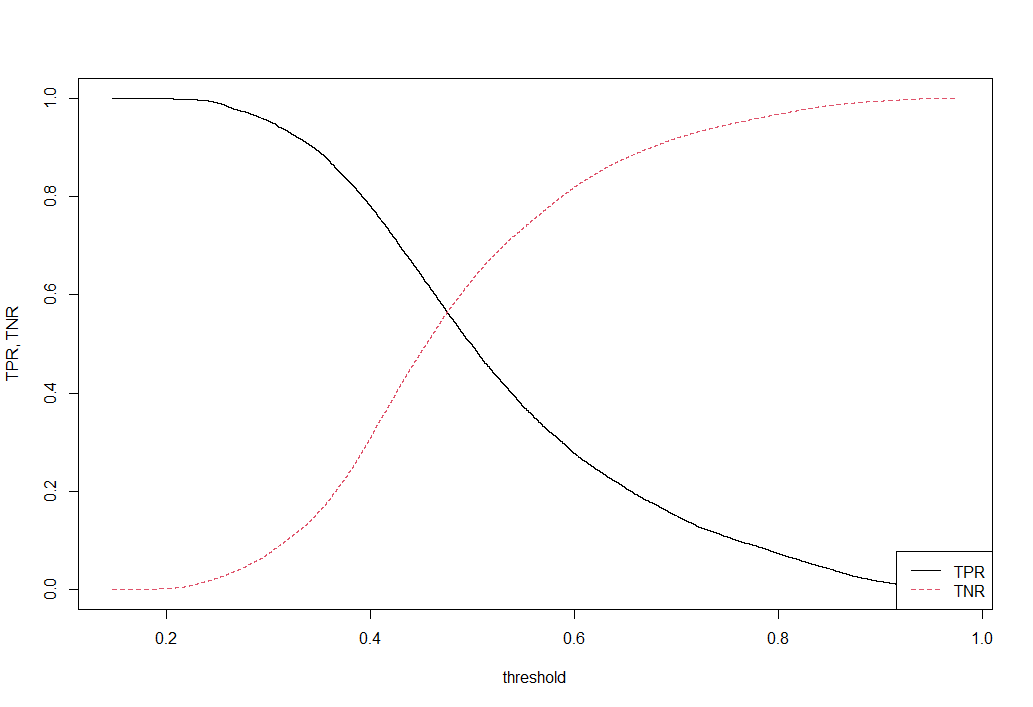 |
| --- | --- |

# Altered morphology

| 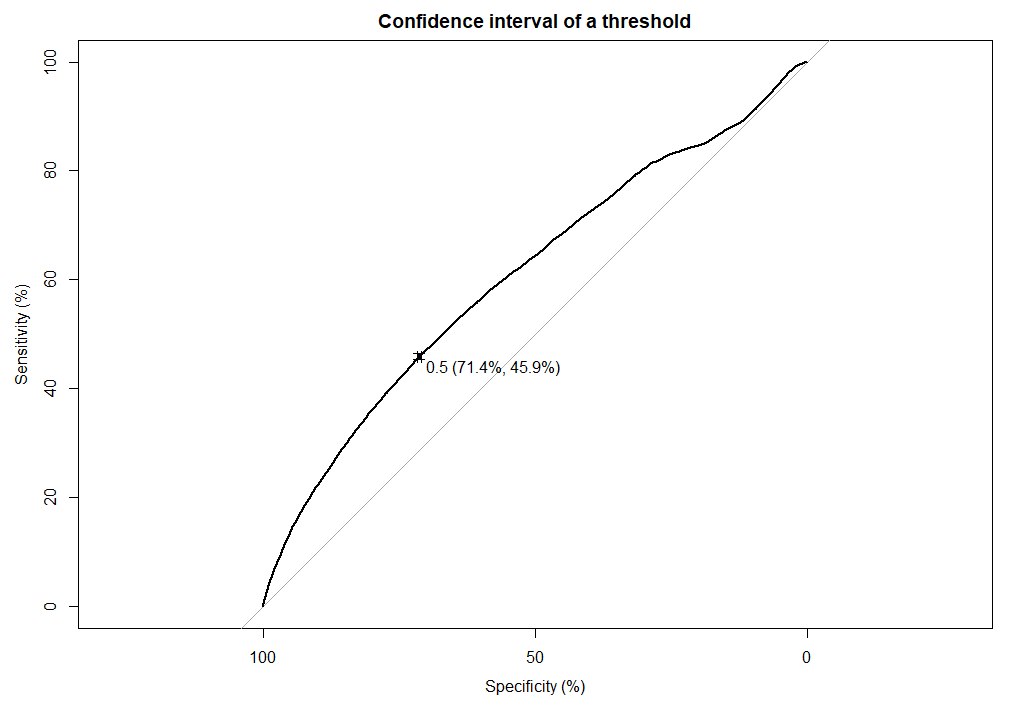 | 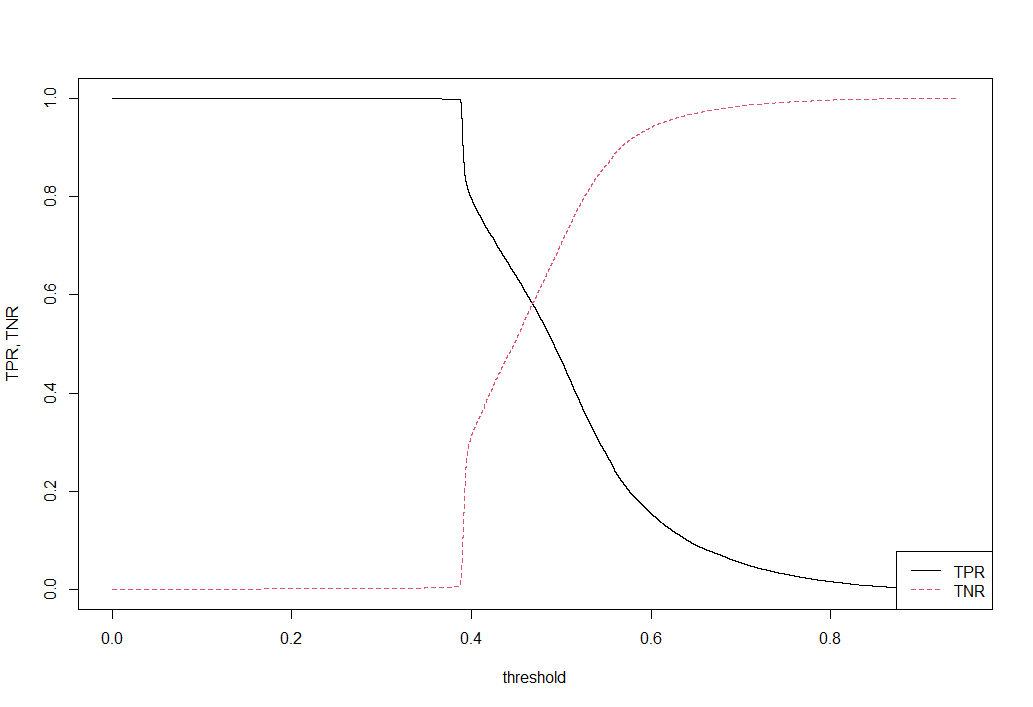 |
| --- | --- |

**No impact**

| 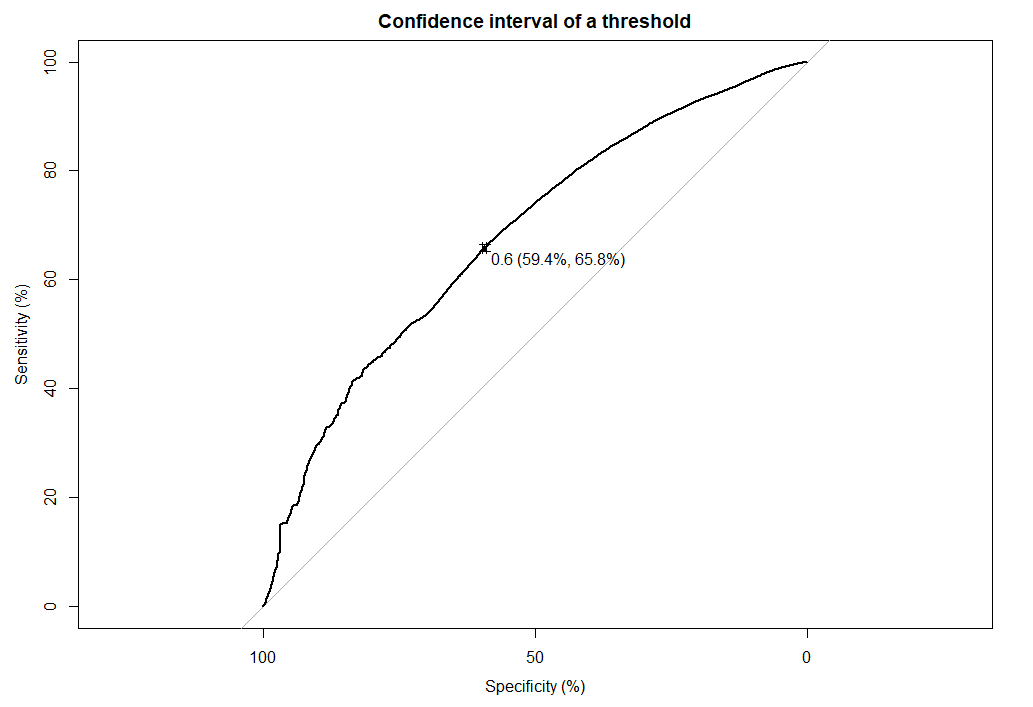 | 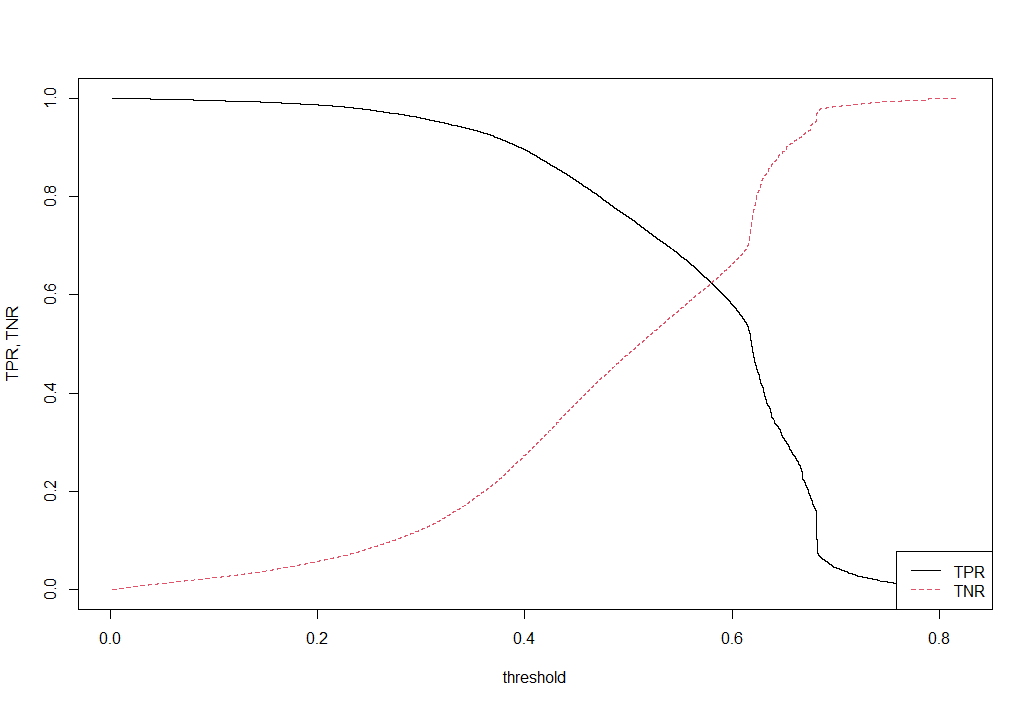 |
| --- | --- |

Table SI2. Results of Testing dataset performance, including sensitivity and specificity when adopting a threshold of 50%. Optimal threshold is the probability where sensitivity and specificity curves cross, i.e. K_C_ is maximum.

|  | Testing dataset | | | | | |
| --- | --- | --- | --- | --- | --- | --- |
|  | Acc | k | Sensitivity | Specificity | AUC | Optimal threshold |
| Failing good ES | 0.64 | 0.3 | 0.56 | 0.75 | 0.68 | 0.52 |
| Nutrient pollution | 0.71 | 0.36 | 0.79 | 0.63 | 0.76 | 0.42 |
| Organic pollution | 0.7 | 0.25 | 0.79 | 0.57 | 0.74 | 0.42 |
| Chemical pollution | 0.41 | -0.19 | 0.58 | 0.64 | 0.61 | 0.43 |
| Altered hydrology | 0.64 | 0.06 | 0.37 | 0.70 | 0.54 | 0.51 |
| Altered morphology | 0.59 | 0.1 | 0.55 | 0.57 | 0.55 | 0.46 |
| No impact | 0.54 | 0.16 | 0.73 | 0.49 | 0.59 | 0.52 |

7) Validation of model against balanced subsets

The estimation of the model skill was made with three validation data subsets (also sampled 100 times):

(A) testing: the model assessment was conducted applying a 10-fold cross validation, leaving out a 10 % of data entries for the model testing;

(B) B<50: a balanced sample that was drawn from the “wb<50” group respecting the same rules as for extraction of the training set. This subset is thus balanced in terms of presences and absences and of country representativeness. However, as it is drawn from the “wb<50” group, the spatial correspondence between geodatasets is weaker than for the testing case;

(C) ALL: all data not used in the training (this is the validation dataset of the manuscript). This group includes all available data, but is imbalanced with respect to presences/absences or country representativeness, and has mixed spatial correspondences.

The median overall accuracy Acc of logistic regressions for the validation testing subsets ranged from 0.58 to 0.69 (Table SI3), K_C_ from 0.16 to 0.38, and AUC from 0.61 to 0.75. These results were largely confirmed in the validation balanced subset B<50, indicating that the regressions were also robust when the spatial overlapping of WISE-CCM2 pairs was less than 50% of the water body. In validation ALL subset (all data except the training sample), performances lowered slightly: the median Acc ranged from 0.55 to 0.73, K_C_ from 0.07 to 0.37, and AUC from 0.56 to 0.79. Overall, model performances could be judged from moderate (chemical pollution, altered hydrology and morphology) to good (ecological status, nutrient and organic pollution). In what follows we look more closely to some specific cases.

Table SI3. Evaluation of the logistic regressions for predicting the probability of failing to achieve good ecological status (ES)or occurrence of nutrient pollution; organic pollution; chemical pollution; altered hydrology; altered morphology; and no impact. Results are reported for three data subsets: testing = 10% of training sample used in 10-fold cross-evaluation; B<50: a balanced sample from the wb<50 group; and ALL: all data except the training sample. For each subset sample size, overall accuracy (Acc), Cohen’s kappa (K_C_), and Area under the Curve (AUC) are reported. The prevalence is the fraction of presences (ones) in the dataset and is reported for the entire abridged dataset (75801 entries, observed prevalence) and for subset ALL. (In the other two cases prevalence is 0.50 by construction.) Reported values are the medians of 100 repetitions of sampling procedure.

|  | | Failing good ES | Nutrient pollution | Organic pollution | Chemical pollution | Altered hydrology | Altered morphology | No impact |
| --- | --- | --- | --- | --- | --- | --- | --- | --- |
| *Observed prevalence* | | *0.55* | *0.27* | *0.17* | *0.43* | *0.16* | *0.37* | *0.28* |
| Testing | sample size | 24578 | 12618 | 7208 | 11234 | 4682 | 13082 | 9232 |
|  | Acc | 0.69 | 0.65 | 0.63 | 0.61 | 0.62 | 0.58 | 0.63 |
|  | K_C_ | 0.38 | 0.30 | 0.26 | 0.21 | 0.24 | 0.16 | 0.26 |
|  | AUC | 0.75 | 0.71 | 0.68 | 0.65 | 0.66 | 0.61 | 0.69 |
| B<50 | sample size | 17102 | 9544 | 5620 | 8018 | 3888 | 8916 | 9240 |
|  | Acc | 0.69 | 0.65 | 0.62 | 0.56 | 0.61 | 0.58 | 0.63 |
|  | K_C_ | 0.38 | 0.31 | 0.24 | 0.11 | 0.21 | 0.16 | 0.27 |
|  | AUC | 0.75 | 0.71 | 0.67 | 0.58 | 0.65 | 0.61 | 0.70 |
| ALL | *Prevalence* | *0.58* | *0.23* | *0.15* | *0.43* | *0.15* | *0.34* | *0.25* |
|  | sample size | 51221 | 63181 | 68591 | 64565 | 71117 | 62717 | 66567 |
|  | Acc | 0.63 | 0.73 | 0.71 | 0.55 | 0.61 | 0.62 | 0.55 |
|  | K_C_ | 0.27 | 0.37 | 0.24 | 0.13 | 0.07 | 0.16 | 0.18 |
|  | AUC | 0.68 | 0.79 | 0.76 | 0.56 | 0.58 | 0.60 | 0.69 |
